# A Reactive Oxygen Species-Responsive Biomimetic Adhesive Hydrogel Mediates Immunoregulation to Effectively Prevent Intrauterine Adhesions

**DOI:** 10.3390/pharmaceutics18060685

**Published:** 2026-05-30

**Authors:** Wanzhen Li, Chenyu Liao, Yuzhen Li, Zijun Lin, Danni Xiao, Gengsheng Ye, Yanjuan Huang, Chunshun Zhao, Shengmiao Cui

**Affiliations:** 1 Guangdong Provincial Key Laboratory of Pharmaceutical Preparations Research and Evaluation, Guangdong Provincial Engineering Center of Topical Precise Drug Delivery System, School of Traditional Chinese Medicine, Guangdong Pharmaceutical University, Guangzhou 510006, China; liwanzhen213@163.com (W.L.); 13726578941@163.com (C.L.); superjunior112005@163.com (Y.L.); 2 School of Pharmaceutical Sciences, Sun Yat-Sen University, Guangzhou 510006, China; linzj29@mail2.sysu.edu.cn (Z.L.); xiaodn@mail2.sysu.edu.cn (D.X.); gengshengye@126.com (G.Y.); huangyj256@mail.sysu.edu.cn (Y.H.)

**Keywords:** intrauterine adhesions, ROS-responsive hydrogel, endometrial regeneration, tannic acid (TA)

## Abstract

**Background:** Intrauterine adhesions, a leading cause of female infertility, frequently recur in 30–62.5% of patients despite hysteroscopic adhesiolysis and adjuvant therapies. Current intrauterine barriers, including injectable hydrogels, often lack sufficient bioactivity and tissue retention, failing to address the underlying pathological inflammation and oxidative stress driving abnormal fibrosis. **Methods:** Herein, we tailored a reactive oxygen species (ROS)-responsive, mussel-inspired adhesive injectable hydrogel (OHA-CP@TA) to intelligently modulate the inflammatory niche and promote normal endometrial regeneration. OHA-CP@TA was fabricated through Schiff base bonds between oxidized hyaluronic acid (OHA) and phenylboronic acid-modified carboxymethyl chitosan (CMCS-PBA), and boronate ester bonds between CMCS-PBA and tannic acid (TA). **Results:** OHA-CP@TA exhibited good mechanical strength, injectability, self-healing, and shear-thinning properties, and importantly, robust and stable adhesion to uterine tissue, overcoming endometrial mucus clearance. It also showed favorable in vivo uterine cavity retention for at least 7 days that covered the critical endometrial repair period. Within the postoperative inflammatory milieu, OHA-CP@TA intelligently released TA in a ROS-dependent manner, which effectively scavenged various ROS and significantly alleviated inflammation, and promoted M1 macrophage polarization into M2 phenotype. This targeted ROS scavenging and immunoregulation inhibited endometrium fibrosis progression, evidenced by downregulation of α-SMA and Col-1, and actively promoted endometrial repair and regeneration, demonstrated by enhanced angiogenesis, increased endometrial thickness, and restoration of glandular numbers. Furthermore, OHA-CP@TA exhibited good biocompatibility, in vivo biodegradability and safety. **Conclusions:** Therefore, OHA-CP@TA represents a promising, clinically translatable strategy for overcoming the limitations of current IUA management.

## 1. Introduction

Infertility has emerged as a non-negligible public health concern, affecting an estimated 15% of the global population [1]. Endometrial damage and the resulting intrauterine adhesions (IUA), characterized by uterine cavity abnormal fibrosis, are considered the most common cause of secondary female infertility [2,3,4]. IUA typically arises from uterine cavity manipulation, particularly induced abortion and curettage, with an incidence of approximately 19–45% [5]. These procedures often damage the endometrium basal layer, leading to endometrial fibrosis, excessive deposition of extracellular matrix (ECM), thin endometrium, and decreased glandular activity, ultimately leading to clinical sequelae such as hypomenorrhea, amenorrhea, recurrent miscarriage, and infertility, profoundly affecting women’s reproductive potential and physical/mental health [3,6,7,8]. Current clinical management of IUA primarily involves hysteroscopic adhesiolysis, supplemented by high-dose estrogen-progesterone regimens [7,9]. However, recurrence rates post-surgery remain unacceptably high, ranging from 30% to 62.5% [10,11], and prolonged high-dose estrogen administration carries undesirable risks to patients [7,9]. Application of physical barriers within the uterine cavity represents another common postoperative adjuvant therapy strategies, which include Intrauterine Device, Foley catheters, uterine balloons, and cross-linked sodium hyaluronate hydrogels [12]. Nevertheless, solid devices (e.g., balloons, catheters) fail to conform adequately to diverse uterine cavity geometries, resulting in poor efficacy at uterine margins [13]. Furthermore, sustained mechanical pressure from these implants may impair endometrial regeneration, induce local inflammation, and carry bacterial colonization risk [14]. In contrast, injectable cross-linked sodium hyaluronate hydrogels (e.g., GongAnkang) that can completely cover every corner of a uterine cavity show great potential for IUA prevention. Unfortunately, clinical evidence indicates that they often yield suboptimal therapeutic efficacy [15,16]. This inadequacy may stem from two critical limitations. First, they primarily function as passive physical barriers, lacking the bioactive properties necessary to modulate an injured endometrium microenvironment [17], such as mitigating oxidative stress and suppressing inflammation, and thereby promoting endometrial repair. Second, they suffer from compromised tissue retention [16], rendering them ineffective during crucial phases of IUA development. Consequently, there is a pressing need for advanced injectable hydrogel systems that integrate robust bioactivity with enhanced tissue-adhesive properties for effective prevention and treatment of IUA.

Persistent or dysregulated inflammation following endometrial injury acts as a central driver of fibrosis, creating a pathological environment that disrupts normal endometrial repair [5]. Excessive activation of inflammatory cells, particularly macrophages, leads to sustained generation of reactive oxygen species (ROS) and pro-inflammatory cytokines (e.g., TNF-α, IL-1β, and IL-6), which activate pro-fibrotic signaling pathways and impair endometrial regeneration, culminating in fibrosis [18]. Consequently, endowing hydrogels with ROS scavenging and anti-inflammation functions are highly desirable for preventing IUA. Importantly, because transient, well-regulated inflammation is essential for debridement and endometrial repair initiation, whereas its persistence drives pathology [5]. Therefore, restoring endometrial homeostasis hinges not on blanket inflammation suppression, but on intelligent, on-demand modulation of the inflammatory response. This necessitates developing smart hydrogel platforms capable of dynamically sensing and adapting to evolving pathological cues in the uterine cavity, but such intelligent, responsive systems remain largely unexplored for IUA prevention.

In addition to bioactivity, achieving interfacial adhesion to endometrial surface is pivotal for functional hydrogels to fully realize their potential in IUA prevention [17]. Reliable interfacial integration of hydrogels ensures in vivo stability and effective biomaterial–tissue crosstalk [17]. The uterine environment poses a specific challenge: copious endometrial mucus production (~3–4 g every 4 h) creates a fluid barrier that efficiently removes non-adherent materials [19]. Hydrogels lacking strong mucoadhesion face rapid detachment, leading to inadequate residence time, suboptimal therapeutic dosage, and crucially, a physical disconnect from the uterine cavity that offset localized bioactivity aimed at preventing scar tissue formation. Furthermore, gaps between non-adhesive implants and tissue surfaces increase the risk of foreign body reactions, which induce fibrous capsule formation on implant surfaces and further impair their biological functions [20]. Consequently, inherent engineering and robust tissue-adhesive properties in therapeutic hydrogels are essential to overcome these delivery obstacles and enhance IUA prevention efficacy.

In recent years, various functional hydrogels have been developed for endometrial repair and IUA prevention. Cai et al. [21] reported an injectable GHD hydrogel based on glycyrrhizic acid (GA) and dopamine-modified hyaluronic acid (HA-DA), which enabled sustained delivery of drugs and growth factors to promote endometrial regeneration. Chen et al. [22] developed a DN hydrogel composed of a crosslinked HA network and a fibrin network formed after PRP activation, which improved PRP retention and sustained the release of PDGF-BB and VEGF. Zheng et al. [23] reported an injectable tHA-tChi hydrogel crosslinked by disulfide bonds for co-delivery of PRP and ADSCs, showing beneficial effects on angiogenesis, endometrial repair, and fertility restoration. Ji et al. [24] further developed a pH-responsive HA-SH hydrogel incorporating peptide-loaded liposomes, enabling lesion-localized release and regulation of macrophage-related fibrotic signaling. These studies have greatly advanced the application of hydrogels in endometrial regeneration, anti-inflammatory regulation, and drug delivery. However, from the perspective of postoperative IUA prevention, it remains challenging to integrate sufficient structural support, prolonged intrauterine retention, and coordinated regulation of inflammation and fibrosis into a single injectable hydrogel system.

**Scheme 1:**
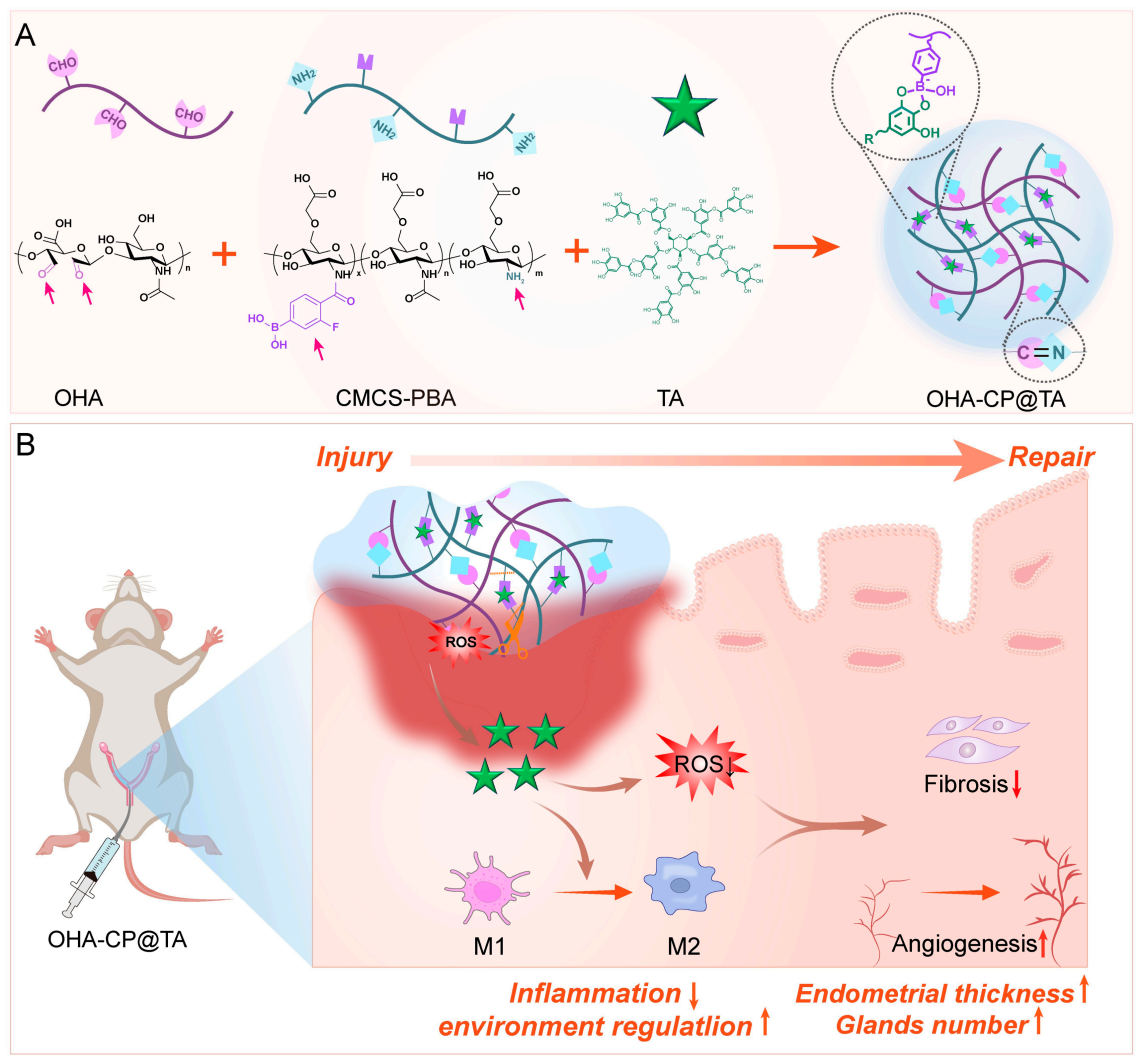
(**A**) Schematic illustration of the preparation of injectable OHA-CP@TA hydrogels. (**B**) Schematic illustration of the mechanism of OHA-CP@TA hydrogels for inflammation environment regulation to prevent IUA and promote endometrial regeneration.

Herein, leveraging the elevated ROS levels in the damaged uterine inflammatory microenvironment, we tailored a ROS-responsive, mussel-inspired biomimetic adhesive injectable hydrogel (OHA-CP@TA) to intelligently modulate inflammation and promote normal endometrial regeneration. Hyaluronic acid (HA) and chitosan (CS), two natural polysaccharide renowned for their good biocompatibility and biodegradability, were chosen as the hydrogel precursor skeleton materials [25,26]. First, oxidized hyaluronic acid (OHA) and 4-carboxy-3-fluorophenylboronic acid (FPBA) modified carboxymethyl chitosan (CMCS-PBA) were synthesized. Then, tannin acid (TA), a natural polyphenolic compound rich in catechol group and with ROS scavenging and anti-inflammation capacities [27,28], was cleverly employed as a hydrogel cross-linker. Subsequently, OHA-CP@TA was fabricated through Schiff base bonds between the amino groups of CMCS-PBA and the aldehyde groups of OHA, and boronate ester bonds between the PBA groups of CMCS-PBA and the catechol groups of TA (Scheme 1A).

The as-designed dual-crosslinking hydrogel exhibited improved mechanical performances to support uterine wall separation and has good self-healing and shear-thinning behavior, which can be easily injected through needles/catheter and rapidly recovers its bulk mechanical strength post-injection, rendering it highly suitable for non-invasive therapeutic applications. Upon injection into the uterine cavity with injured endometrium, OHA-CP@TA hydrogel formed stable interfacial adhesion to the entire corner of uterine tissue via covalent and physical interactions through a mussel-inspired biomimetic adhesive mechanism owing to the catechol group in TA. Additionally, the covalent bonding between the aldehyde groups in hydrogel with tissue further enhanced the interfacial adhesion strength. Then, TA was intelligently released in a ROS level-dependent manner within the inflammatory microenvironment, which not only effectively scavenge various ROS, but also alleviate inflammatory responses and promote M1 macrophage polarization into M2 phenotype. With the attenuation of inflammation and ROS, the fibrosis progression was inhibited and endometrial repair and regeneration occurred. This was manifested by the downregulation of α-SMA and COI-1, and the increased angiogenesis, endometrial thickness and glandular numbers. Furthermore, this hydrogel showed favorable in vivo retention that covered the critical endometrial repair period, and exhibited excellent biocompatibility, biodegradability and in vivo safety. Collectively, these findings demonstrated that by providing a sustained physical barrier while simultaneously modulating the hostile inflammatory microenvironment, OHA-CP@TA effectively prevented IUA and promoted functional endometrial repair. We propose this hydrogel not only offers a promising approach for IUA prevention, but also presents a clinically translatable strategy for endometrial regeneration and repair.

## 2. Materials and Methods

### 2.1. Materials, Cell Lines and Animals

Materials: Sodium hyaluronate (HA, M_W_: 50 kDa) was purchased from Bloomage Biotechnology Corporation Limited (Jinan, China). Carboxymethyl chitosan (CMCS, M_W_: 100–200 kDa) and 2,2-Diphenyl-1-picrylhydrazyl (DPPH) were purchased from Macklin Biochemical Co., Ltd. (Shanghai, China). 2′,7′-dichlorofluorescent yellow diacetate (DCFH-DA) was obtained from MedChemExpress LLC (Monmouth Junction, NJ, USA). Tannin (TA), 1-Ethyl-3-(3-dimethylaminopropyl) carbodiimide hydrochloride (EDCI), N-Hydroxysuccinimide (NHS), and 4-carboxy-3-fluorophenylboronic acid (FPBA) were purchased from Bide Pharmatech Co., Ltd. (Shanghai, China). Fluorescein diacetate (FDA), NaIO_4_, and ascorbic acid (Vc) were purchased from Aladdin Reagents Co., Ltd. (Shanghai, China). SYBR Green Premix Pro Taq HS qPCR Kit, Trizol, and Evo M-MLV Reverse Transcription Kit II were purchased from Accurate Biotechnology Co. Ltd. (Changsha, China). 3-(4,5-dimethyl-2-thiazolyl)-2,5-diphenyl-2H-tetrazolium bromide (MTT), LPS, Trypsin-EDTA, and 4′,6-diamidino-2-phenylindole (DAPI) were purchased from Sigma-Aldrich Co. LLC (St. Louis, MO, USA). Anti-CD16/32, anti-CD86-PC7, and anti-CD206-PE were purchased from eBiosciences (Hatfield, UK). The primers used for RT-PCR were obtained from Sangon Biotech Co., Ltd. (Shanghai, China). Interleukin-4 (IL-4) and interferon-γ (IFN-γ) were purchased from PeproTech (Cranbury, NJ, USA).

Cell lines: RAW 264.7 cells and L929 cells were procured from the Laboratory Animal Center of Sun Yat-sen University (Guangzhou, China), both cells were maintained in Dulbecco’s Modified Eagle Medium (DMEM, Gibco, Waltham, MA, USA) supplemented with 10% fetal bovine serum (FBS, Gibco) and 1% (*v*/*v*) penicillin–streptomycin (Sigma-Aldrich). Isolated primary rat endometrial stromal cells (rEnSCs) were cultured in DMEM/F12 (Hyclone, Logan, UT, USA) supplemented with 10% (*v*/*v*) FBS and 1% (*v*/*v*) penicillin–streptomycin. All cells were cultured in an incubator maintained at 37 °C with 5% CO_2_ and 95% air.

Animals: Female Sprague-Dawley rats (180–220 g, 8–10 week) were acquired from the Laboratory Animal Center of Sun Yat-sen University. All experimental procedures involving animals were conducted in strict compliance with the guidelines and regulations approved by the Institutional Animal Care and Use Committee (SYSU-IACUC-2025-001954) of Sun Yat-sen University.

### 2.2. Synthesis and Characterization of OHA

OHA was prepared based on our published method [29]. Briefly, 2.00 g of HA was dissolved in 50 mL of deionized water. Subsequently, NaIO_4_ (1.5 g, 7.5 mmol) dissolved in 10 mL of deionized was added dropwise to the HA solution. The reaction was conducted in the dark for 6 h. Thereafter, 2 mL ethylene glycol was added dropwise and reacted for 2 h to quench the reaction. The reaction solution was dialyzed against pure water for purification and lyophilized to obtain OHA. The structure of OHA was characterized by ^1^H NMR Fourier transform infrared spectroscopy (FTIR, VERTEX 70, Bruker, Waltham, MA, USA), FT-IR (Nicolet 6700, Thermo Fisher, Waltham, MA, USA), and UV-vis spectroscopy (UV2600, Kyoto, Japan).

### 2.3. Synthesis and Characterization of CMCS-PBA

FPBA (83.16 mg) was dissolved in 5 mL of DMSO, followed by the addition of EDCI (103.97 mg) and NHS (62.42 mg). The mixture was stirred at room temperature for 2 h to activate the carboxyl groups. Meanwhile, CMCS (1 g) was dissolved in 100 mL of deionized water, and the activated FPBA solution was added dropwise and reacted for 24 h. The reaction solution was dialyzed against 95% ethanol/water (1:5, *v*/*v*) for 1 day, and then against pure water for 2 days. CMCS-PBA was obtained by lyophilization, and the structure was analyzed by ^1^H NMR, FT-IR, and UV-vis spectroscopy.

First, a series of FPBA standard solutions with gradient concentrations were prepared, and their absorbance at 232 nm was measured to establish the FPBA calibration curve. Then, lyophilized CMCS-PBA samples were dissolved and diluted to fall within the linear range of the standard curve. The absorbance of each sample was measured in triplicate. The corresponding FPBA concentration was calculated from the calibration curve, and the FPBA content in CMCS-PBA was determined using the following equation:

$$Wt(\%)=\frac{C_{X}\times V}{m_{sample}}\times 100\%$$

Cx is the FPBA concentration calculated from the standard curve; V is the total volume of the sample solution, and $m_{sample}$ is the mass of the lyophilized CMCS-PBA sample.

### 2.4. Fabrication and Characterization of OHA-CP@TA Hydrogel

Hydrogel precursor materials were prepared in pH 7.4 PBS buffer. OHA was first mixed with CMCS-FPBA, and then immediately vortexed with TA solution at room temperature to prepare the OHA-CP@TA hydrogel. Initial formulation screening for hydrogel optimization was conducted with a rotational rheometer (MCR302e, Anton Paar GmbH, Graz, Austria), following the formulations detailed in Table 1. The rheological parameters were set as follows: the measuring plate was a cone-plate PP-08, the gap was 1 mm, the test temperature was 25 °C, and the air pressure was 5 bar. Time sweep tests at a duration of 3 min were conducted at different hydrogel formulations at 1 Hz frequency and 1% strain.

Based on the mechanical strength screening, 0.5 mL of OHA-CP@TA hydrogel was prepared by first mixing OHA (30 mg/mL, 100 μL) with CMCS-FPBA (40 mg/mL, 350 μL), and then immediately vortexed with TA solution (2.5 mg/mL, 50 μL) at room temperature. For hydrogels with other volumes or different formulations, the same procedure was followed, with the amounts of each component adjusted according to the formulations listed in Table 1 to ensure that the final mass concentrations of OHA, CMCS-FPBA, and TA were consistent with the corresponding formulation. Blank OHA-CP hydrogels were prepared using the same method without adding TA. The gelation mechanism of the lyophilized hydrogels was verified by FT-IR. The microscopic morphology of OHA-CP@TA hydrogels with or without H_2_O_2_ treatment was imaged by scanning electron microscope (SEM, Zeiss, Jena, Germany). The injectable performance of OHA-CP@TA hydrogel was evaluated via a 22 G needle or catheter-equipped syringe.

### 2.5. Rheological and Self-Healing Properties of OHA-CP@TA Hydrogels

The rheological properties of hydrogels were analyzed by rotational rheometer using the same parameters as above. Strain sweep tests (1 Hz) were conducted at 0.1–100% strain range to determine the linear viscoelastic region. Frequency sweep tests (1% strain) were performed on 0.1 Hz to 100 Hz. Shear-rate sweep tests were monitored at the shear-rate range of 0.1–1000 s^−1^ to investigate shear-thinning behaviors.

The viscosities of TA (2.5 mg/mL), OHA (30 mg/mL), and CMCS-FPBA (40 mg/mL) precursor solutions were measured. Measurements were performed using a CP50 cone-plate geometry with a gap of 0.1 mm over a shear-rate range of 10–1000 s^−1^.

Subsequently, the self-healing capacities of hydrogels were explored by using cyclic tests involving alternating strain and shear-rate over three successive cycles, respectively. Specifically, step-strain cycling tests were performed by switching between 1% and 100% strain at a constant frequency of 1 Hz, while step-shear-rate cycling tests alternated between 1 s^−1^ and 100 s^−1^. For macroscopic self-healing, two OHA-CP@TA hydrogels were split into two parts, the unstained and xylenol orange-labeled parts were taken into contact for a specific period, then the junction was examined after manipulating it using tweezers.

### 2.6. Tissue Adhesion of OHA-CP@TA Hydrogels

To investigate the tissue adhesion of OHA-CP@TA hydrogels towards endometrial tissue, 200 μL of hydrogel was sandwiched between two rat endometrial slices (overlapping area: 0.0050 m^2^). The upper endometrial slice was adhered to the rheometer’s rotor using 502 glue, while the lower endometrial slice was fixed on the rheometer’s measurement platform. Adhesion force (N) was measured at an upward speed of 100 μm/s for 2 min. Tissue adhesion strength (kPa) was calculated by maximum adhesion force (N) divided by the overlapping area (m^2^). Measurements were repeated 3 times.

### 2.7. In Vitro Swelling and Degradation of OHA-CP@TA Hydrogels

The water-uptake capacity of lyophilized OHA-CP and OHA-CP@TA hydrogels was evaluated by immersing dried hydrogel samples in uterine-simulating fluid (*n* = 3). At 1, 2, 3, 5, 7, and 28 h, hydrogels were retrieved, surface liquid was adsorbed with filter paper and weighed. The swelling calculation formula is as follows:

$$\mathrm{Swelling}\;(\%)\;=\;\frac{\left(W_{t}-W_{0}\right)}{W_{0}}\times 100\%$$

where W_t_ is the weight of the hydrogel at the corresponding time, and W_0_ is the initial dry weight of the hydrogel. Measurements were repeated 3 times.

The in vitro degradation behavior was assessed by immersing OHA-CP@TA hydrogels in uterine-simulating fluid. Each hydrogel was first swollen in 2 mL of uterine simulant for 48 h. The surface liquid was gently removed with filter paper, and the mass of the swollen hydrogels was recorded as W_0_ (degradation initial mass). The swollen hydrogels were continuously incubated, and their masse (W_t_) was measured at 12, 24, 48, 72, 96, 120, 144, 168, 192, 240, 288, and 336 h (surface liquid absorbed before weighing). The in vitro degradation rate was calculated as follows: 


$$\mathrm{Residual}\;\mathrm{weight}\;\mathrm{precentage}\;(\%)\;=\;\frac{W_{t}}{W_{0}}\times 100\%$$

**Table 2:** Compositions of simulated uterine fluid (g/L) [30].

NaCl	KCl	CaCl_2_	NaHCO_3_	Glucose	NaH_2_PO_4_·2H_2_O
4.79	0.224	0.167	0.25	0.50	0.072

### 2.8. DPPH Radical Scavenging Assay

The DPPH radical scavenging activity of free TA and hydrogel was evaluated by mixing TA and Vc (final concentrations of 2.5, 5, 10, 20, 40, and 80 μg/mL), or OHA-CP and OHA-CP@TA hydrogels (2.5, 5, 7.5 and 10 mg/mL) with DPPH methanol solution (0.2 mM). The mixtures were incubated in the dark at an ambient temperature for 40 min. Subsequently, the absorbance of each sample at 517 nm was measured, and the DPPH radical scavenging rate was calculated by the following formula:

$$\mathrm{DPPH}\;scavenging(\%)=(1-\frac{A_{sample}-A_{blank}}{A_{control}})\times 100\%$$

where A_control_, A_blank,_ and A_sample_ represented the absorbances of the control (DPPH), the blank (drugs or hydrogel), and the samples (drugs or hydrogel + DPPH), respectively. Measurements were repeated 3 times.

### 2.9. ABTS+ Radical Scavenging Assay

ABTS radicals were generated by mixing 25 mL 7.4 mM ABTS aqueous solution with 25 mL 3.7 mM potassium persulfate aqueous solution, followed by incubation in the dark for 12 h. This stock was diluted to a 0.06% (*v*/*v*) ABTS radicals working solution. Thereafter, different concentrations of TA and Vc (0.25, 0.5, 1.25, 2.5, 5, 8, μg/mL), or OHA-CP and OHA-CP@TA hydrogels (0.25, 0.5, 1.25, 2.5 and 7.5 mg/mL) were incubated with the working solution in the dark for 30 min, and the absorbance at 734 nm was measured via a microplate reader. The ABTS radical scavenging rate was calculated as follows:

$$\mathrm{ABTS}\;\mathrm{radical}\;scavenging(\%)=(1-\frac{A_{sample}-A_{blank}}{A_{control}})\times 100\%$$

where A_control_, A_blank,_ and A_sample_ denoted the absorbances of the control (ABTS), the blank (drugs or hydrogel), and the samples (drugs or hydrogel + ABTS), respectively. Measurements were repeated 3 times.

### 2.10. Hydroxyl Radical (•OH) Scavenging Assay

The •OH scavenging abilities were evaluated using TMB as a probe. Briefly, TA and Vc (final concentration: 20, 50, 75, 100, 200, and 300 μg/mL), or OHA-CP and OHA-CP@TA hydrogels (final concentration: 2.5, 5, 10, and 25 mg/mL) prepared in a pH 4.0 CH_3_COOH-CH_3_COONa solution were incubated with solutions containing FeCl_2_ (5 mM), H_2_O_2_ (5 mM), and TMB (25 μg/mL) in the dark for 30 min. The absorbance of each sample at 650 nm was measured via a microplate reader. The •OH radical scavenging rate was calculated as follows:

$$•OH\;scavenging(\%)=(1-\frac{A_{sample}-A_{blank}}{A_{control}})\times 100\%$$

where A_control_, A_blank_, and A_sample_ denoted the absorbances of the control (TMB + Fenton), the blank (drugs or hydrogel + TMB + FeCl_2_), and the samples (drugs or hydrogel + TMB + Fenton), respectively. Measurements were repeated 3 times.

### 2.11. In Vitro ROS-Responsive Drug Release

To investigate ROS-responsive drug release, 1 mL of OHA-CP@TA hydrogel was placed in a dialysis bag (MWCO: 3500 Da) and immersed in 10 mL of PBS (pH 7.4) containing different concentrations of H_2_O_2_ (0 μM, 100 μM, and 300 μM). They were kept in a shaker, at designated time points, 1 mL of release medium was collected and replenished with 1 mL fresh medium. The concentration of TA in release medium was determined via UV-vis spectrophotometry at 307 nm, and the cumulative release rate of TA was calculated.

In addition, because H_2_O_2_ may affect the oxidation state of polyphenolic compounds, TA standard curves were established in release media containing the corresponding H_2_O_2_ concentrations to ensure comparability among different release conditions.

### 2.12. Isolation and Culture of rEnSCs

Endometrial tissue was isolated from Female Sprague-Dawley rats (8–10 weeks), then washed, and the adherent adipose tissues were removed. Subsequently, the endometrial tissue was longitudinally dissected and digested with 1% trypsin (5 mL/uterus) at 37 °C for 30 min, followed by vertexing for 10 s. The mixture was filtered through a 70 μm cell strainer and then rinsed. The filtrate was centrifuged, and the cells were cultured in a cell plate. When the cells confluence reached 80–90%, they were treated with Trypsin-EDTA for serial passaging. Following propagation to passages 3–5, the cells were used for experiments or cryopreserved.

### 2.13. Cytocompatibility Evaluation

The cytocompatibility of hydrogel precursor materials and extracts were investigated in RAW 264.7 cells, L929 cells and rEnSCs using the MTT assay. RAW 264.7 cells and L929 cells seeded in 96-well plates were incubated with different concentrations of precursor materials (OHA, CMCS-PBA) and incubated for 24 h prior to MTT assay. To investigate the cytocompatibility of hydrogels, RAW 264.7 cells, L929 cells and rEnSCs seeded in 12-well plates were incubated with OHA-CP and OHA-CP@TA hydrogels (25 μL/2 mL culture medium) using a Transwell^®^ system, for 24 h or 72 h, respectively. After treatment, MTT solution (5 mg/mL) was added, and the cells were treated for an additional 4 h. After fully dissolving the formazan with DMSO, the absorbance of each well at 490 nm was measured using a microplate reader (ELX800, Bio-Tek, Winooski, VT, USA) to quantify the cell viability.

Live/Dead cell staining was further conducted, after the cells were treated with hydrogels for 24 h or 72 h, cells were washed, then stained with FDA (10 μg/mL) and PI solution (5 μg/mL) for 15 min, washed and then imaged by a cell imaging system (BDS400, Chongqing, China).

### 2.14. Hemocompatibility Evaluation

Hemocompatibility of the hydrogels was assessed using an in vitro hemolysis test. Briefly, hydrogel samples (50 μL and 100 μL) were mixed with 1 mL of 5% (*v*/*v*) erythrocyte suspension. Water and saline served as positive and negative controls, respectively. All mixtures were incubated at 37 °C for 2 h, and then centrifuged at 2000 rpm for 10 min, the absorbance of the supernatant was measured at 540 nm using a microplate reader. Hemolysis rate was calculated as: 


$$\mathrm{Hemolysis}\;\mathrm{rate}\;(\%)\;=\;\left(\frac{A_{sample}-A_{negative}}{A_{positive}-A_{negative}}\right)\text{×}100\%$$

### 2.15. Intracellular ROS Scavenging, Anti-Inflammatory and Macrophage Phenotype Regulation

Hydrogel extracts were obtained by incubating hydrogels (25 μL/mL culture medium) with fresh cell culture medium in an incubator for 24 h and filtered (0.22 μm). The concentration of TA in extract was detected by UV-vis spectrophotometer.

The intracellular ROS scavenging of OHA-CP@TA hydrogels was assessed using DCFH-DA as a probe. RAW 264.7 cells and rEnSCs cells seeded in 12-well plates respectively were treated with 200 μM H_2_O_2_ to establish an oxidative stress model, with untreated cells served as control. H_2_O_2_ treated cells were incubated with hydrogel extract. After that, cells were washed and treated with DCFH-DA (10 μM) for 30 min. For flow cytometry (CytoFLEX S, Beckman Coulter, Brea, CA, USA) detection, cells were washed, harvested and then immediately detected. For laser scanning confocal microscope (LSCM, FV3000, Olympus, Tokyo, Japan) imaging, cells were washed, fixed with 4% paraformaldehyde, stained with DAPI, and then imaged.

Next, the anti-inflammatory effect was further investigated. RAW 264.7 cells were seeded in 6-well plates (2 × 10^5^ cells/well), and incubated with 1 μg/mL LPS plus 40 ng/mL IFN-γ overnight to generate a pro-inflammatory M1 phenotype. M2 phenotype polarization was induced by overnight incubation with 40 ng/mL IL-4. Untreated macrophages were served as M0. Subsequently, the culture medium of M1 macrophages was replaced with a different concentration of free TA or hydrogel extracts with equivalent TA concentrations of 125 nM, respectively. After 24 h of treatment, cells were collected, and total RNA was extracted using a column-based RNA extraction method. RNA concentration and purity were determined using a NanoDrop spectrophotometer, with A260/A280 values between 1.8 and 2.0. The extracted RNA was treated with DNase I to remove genomic DNA, and cDNA was synthesized using the Evo M-MLV Reverse Transcription Kit II. RT-qPCR was performed on a LightCycler II PCR system using the SYBR Green Premix Pro Taq HS qPCR Kit. The relative mRNA expression levels of TNF-α, iNOS, and IL-6 were calculated using the 2^−ΔΔCT^ method and normalized to GAPDH. To evaluate macrophage polarization, cells subjected to the same treatment schedule were collected, incubated with anti-CD16/32 to block Fc receptors, and then stained with anti-CD86-PC7 and anti-CD206-PE antibodies for flow cytometry analysis.

### 2.16. In Vivo Retention, Degradation and Safety Evaluation

To investigate in vivo retention in uterine tissue, near-infrared II fluorescence imaging (NIRF-II) was performed on Cy7.5-labeled commercial HA hydrogel (GongAnkang) and OHA-CP@TA hydrogels, using an NIRF-II small animal in vivo imaging system (NIR-II-ST, Shanghai, China, Digi-united Biotechnology, Ex = 808 nm, Em = 850 nm). Briefly, 100 μL of Cy7.5-labeled hydrogels (Cy7.5 dye 5 μg/rat) were injected into the left uterine cavity with a syringe equipped with a catheter (*n* = 3). The rats were subjected to in vivo imaging at the predetermined time points (0, 3, 7, and 14 days). The acquired fluorescence images were processed with IMAGEBLOCKS software, and the relative fluorescence area was quantitatively analyzed using Image J software.

To investigate in vivo degradation behavior, 100 μL of OHA-CP@TA hydrogel was subcutaneously injected into the backs of the mice. The residual area of the injected hydrogel was dynamically tracked on days 0, 3, 7, and 14 utilizing an MS400 ultrasound imaging system (38 MHz).

To investigate the in vivo safety, plasma samples from the Sham, HA and OHA-CP@TA groups were collected, biochemical indicators, including alanine aminotransferase (ALT), aspartate aminotransferase (AST), blood urea nitrogen (BUN) and creatinine (CREA) were detected. In addition, hearts, livers, spleens, lungs, and kidneys were harvested, fixed in 4% paraformaldehyde, embedded into paraffin, and sectioned for H&E staining.

### 2.17. Animal Model Establishment and Treatment

To establish a rat model of IUA, mature female SD rats (8–10 weeks old, weighing 180–220 g) were utilized and confirmed to have a regular estrous cycle. After the female SD rats were anesthetized, the abdominal hair was shaved. A 2–3 cm midline abdominal incision was made to expose the “Y-shaped” uterus in a sterile environment. Subsequently, a 0.5 cm lateral incision was created on one side of the uterus intended for modeling. A pre-sterilized homemade endometrial scraper was then inserted into the uterine lumen, and mechanical damage was induced by gently scraping the inner uterine wall in a back-and-forth motion, ensuring that the endometrial epithelium and superficial stroma were thoroughly removed.

Subsequently, 100 μL of commercial HA hydrogel (GongAnkang), OHA-CP and OHA-CP@TA hydrogels were injected into the uterine cavity with a syringe equipped with a rat-tail dropper extension. The sham group underwent uterus incision and suturing only, whereas the model group received endometrium injury without treatment. Finally, surgical incisions were sutured in layers using surgical sutures, and antibiotics were applied locally to prevent infection.

Two weeks after surgery, rats were humanely euthanized. Intact uterine tissues were harvested and photographed, with one part immediately fixed in 4% paraformaldehyde and another part frozen at −80 °C for subsequent mechanism evaluation. The fixed specimens were processed into paraffin for sectioning, and histological assessments were conducted using hematoxylin-eosin (H&E) staining and Masson’s trichrome staining.

### 2.18. In Vivo Assessment of Endometrial Repair Mechanisms

Briefly, frozen uterine tissues (20–30 mg) were homogenized on ice using a multi-functional homogenizer, and 1 mL Trizol reagent was added to extract total RNA. Isolated RNA was quantified via NanoDrop (A260/A280: 1.8–2.0) and treated with DNase I (Takara) to eliminate genomic DNA. Complementary DNA (cDNA) was synthesized using Evo M-MLV Reverse Transcription Kit II. RT-qPCR was performed on a PCR instrument (Roche, lightCycler II, Mannheim, Germany) using SYBR Green Premix Pro Taq HS qPCR Kit. Relative mRNA expression (iNOS, IL-6, Arg-1, TGF-β1, COI-1, α-SMA) was calculated via the 2^−ΔΔCT^ method and normalized to GAPDH. Gene-specific primer sequences are listed in Table S1. Additionally, immunohistochemical staining for CD31 and α-SMA of the endometrium was also conducted.

### 2.19. Statistical Analysis

All quantitative data are presented as mean ± standard deviation (SD). Multiple group comparisons were analyzed by one-way ANOVA using Origin software(2020). Statistical significance was defined as: n.s., no significant difference; * *p* < 0.05 (significant); ** *p* < 0.01 and *** *p* < 0.001 (highly significant).

## 3. Results and Discussion

### 3.1. Synthesis and Characterization of OHA and CMCS-PBA Conjugate

**Figure 1:**
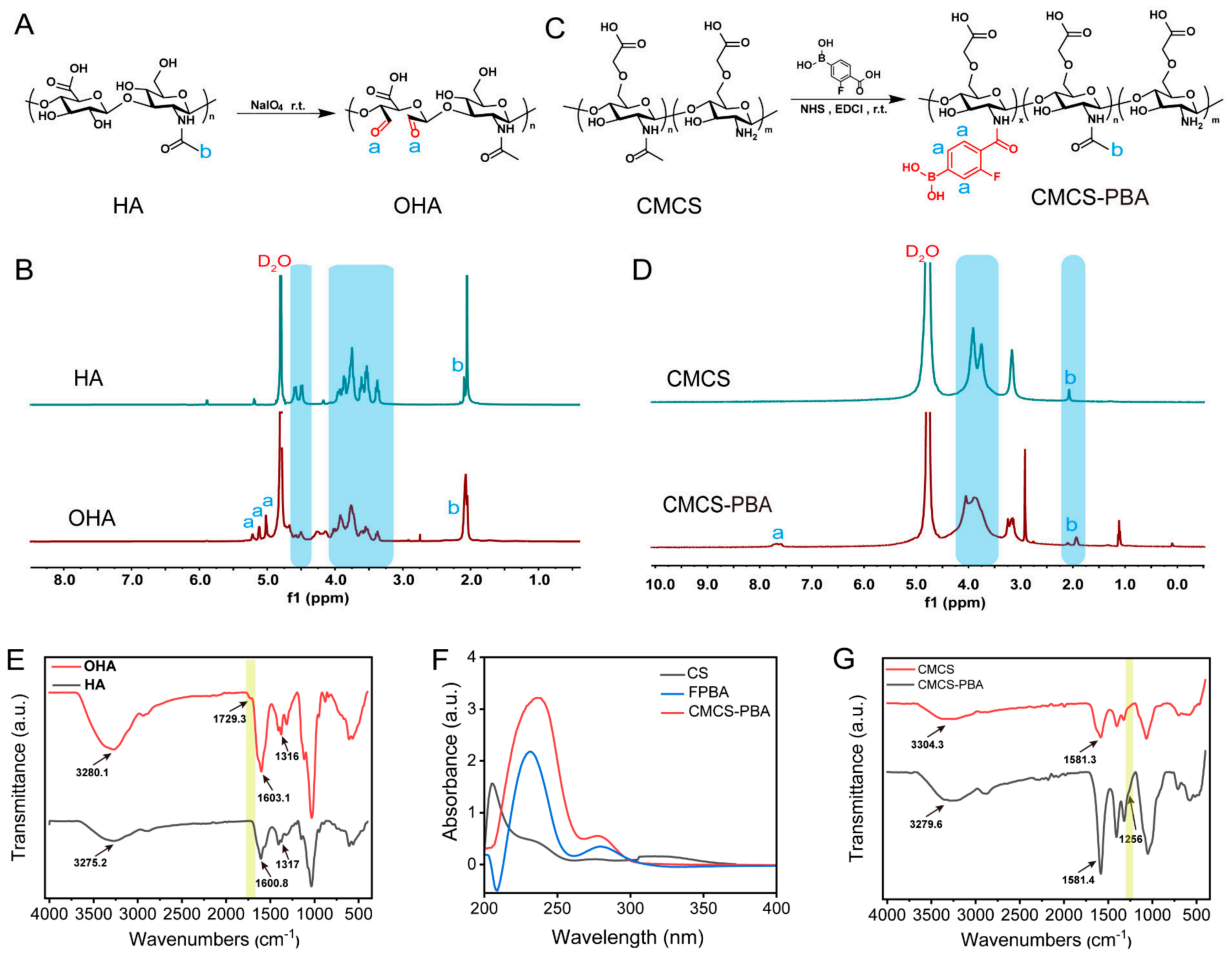
(**A**) The synthesis route and (**B**) ^1^H NMR spectra of OHA. (**C**) The synthesis route and (**D**) ^1^H NMR spectra of CMCS-PBA. (**E**) The FT-IR spectrum of OHA. (**F**) The UV-vis spectrum and (**G**) FT-IR spectrum of CMCS-PBA.

OHA was synthesized via oxidation of the vicinal dihydroxyl groups on HA using NaIO_4_ (Figure 1A). When comparing the ^1^H NMR spectrum of OHA with HA, OHA exhibited distinct new proton signals attributed to aldehyde groups at chemical shifts of 4.89, 4.99, and 5.10 ppm (peak a) (Figure 1B). FT-IR further validated OHA synthesis, as its spectrum displayed a characteristic absorption peak for aldehyde groups at about 1729 cm^−1^ (Figure 1E). Additionally, the oxidation degree of OHA was determined to be approximately 30%.

CMCS-PBA was synthesized through an amidation reaction between the amino groups of CMCS and the carboxyl groups of FPBA (Figure 1C). The ^1^H NMR spectrum of CMCS-PBA revealed distinct characteristic peaks at approximately 7.6~7.7 ppm (peak a) (Figure 1D), which corresponded to the protons on the benzene ring of the PBA. UV-Vis analysis of CMCS-PBA showed characteristic absorption peaks at 236 nm and 278 nm, which corresponded to PBA (Figure 1F). The FT-IR spectrum of CMCS-PBA exhibited a characteristic B-O absorption peak at 1256 cm^−1^ (Figure 1G). The above results confirmed the successful synthesize of CMCS-PBA. The average grafting degree of FPBA in CMCS-PBA was calculated to be 9.01%.

### 3.2. Fabrication and Characterization of OHA −CP@TA Hydrogel

OHA-CP@TA hydrogel was prepared by first mixing OHA with CMCS-PBA, and then immediately mixing this with TA at room temperature. To optimize hydrogel formulations, we investigated the effects of the mass concentrations of OHA, CMCM-PBA and TA on the mechanical strength of hydrogel (Table 1). Based on preliminary experiments, representative hydrogel formulations were selected for further screening. The gelation time should be short enough for rapid in situ formation, but not so rapid as to compromise mixing, injection, and handling. The hydrogel should also possess an adequate storage modulus to maintain structural stability within the uterine cavity. In addition, excessive OHA may leave more unreacted aldehyde groups, increasing the potential risk of cellular or tissue irritation, whereas high polymer concentrations may increase viscosity and impair injectability. Therefore, we first identified a suitable concentration range for forming stable injectable hydrogels through preliminary experiments, and then selected the six representative formulations listed in Table 1 for rheological screening.

**Figure 2:**
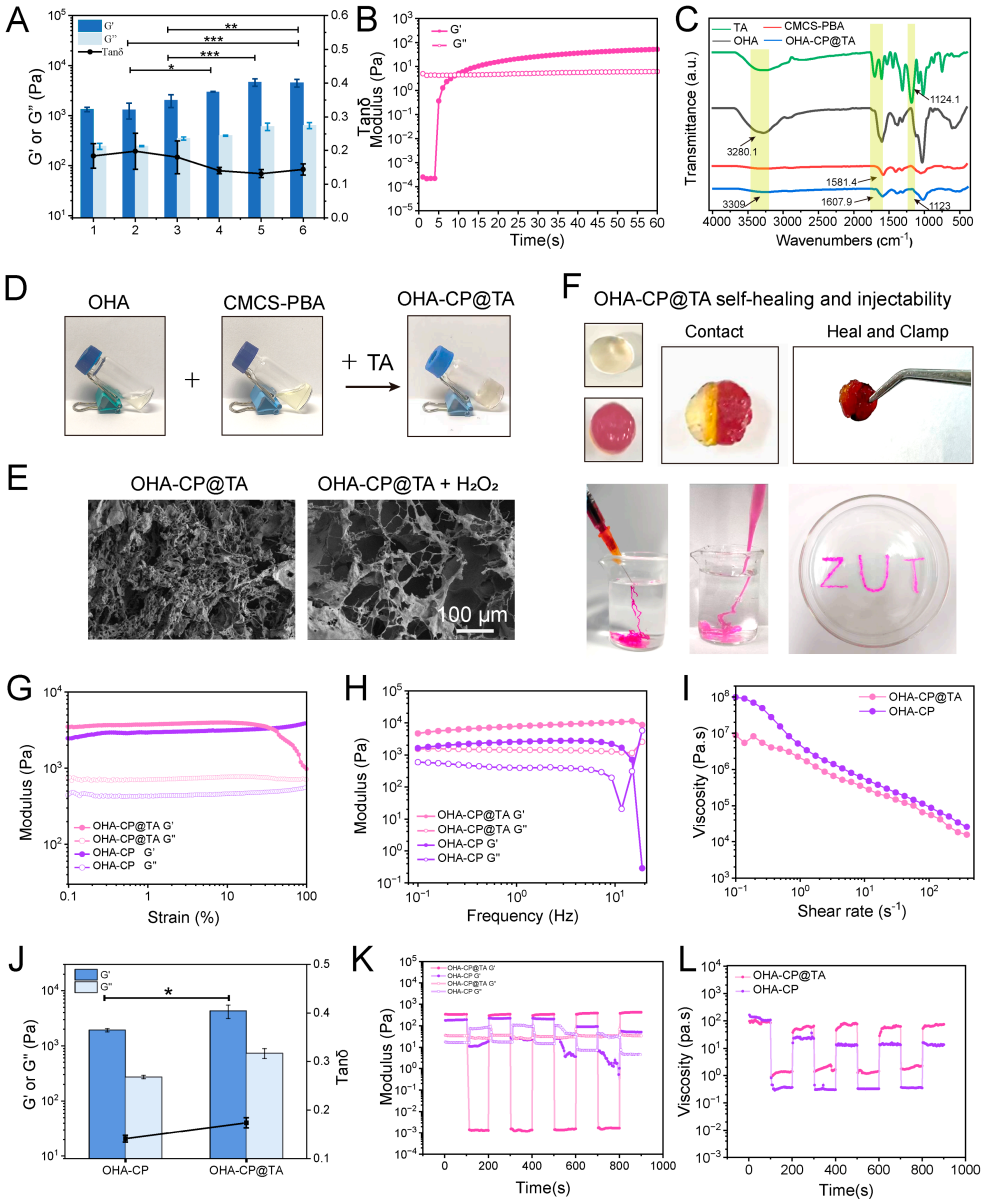
(**A**) Time sweep tests of G′, G″ and Tan δ of different hydrogel formulations. (*n* = 3) (**B**) Gelation time of OHA−CP@TA hydrogel detected by rheometer. (**C**) The FT-IR spectrum of OHA-CP@TA hydrogel. (**D**) The gelation process and (**E**) SEM image of OHA-CP@TA hydrogel with or without H_2_O_2_ treatment. (**F**) Macroscopic observation of self-healing and injectability on OHA-CP@TA hydrogel. (**G**) Strain sweep tests, (**H**) frequency sweep tests, (**I**) shear-rate sweep tests and (**J**) time sweep tests of OHA-CP and OHA-CP@TA hydrogels. (*n* = 3) (**K**) Step-strain tests and (**L**) step-shear-rate tests of OHA-CP@TA hydrogel. * *p* < 0.05, ** *p* < 0.01, *** *p* < 0.001.

Mechanical strength was quantified by measuring the storage modulus (G′) and loss modulus (G″) via a rotational rheometer, and Tan δ were calculated. Tan δ values of the six hydrogels were around 0.2, suggesting they have high viscoelasticity (Figure 2A). As the mass concentrations of CMCS-PBA, PBA and TA increased, the G′ values increased accordingly. The higher G′ values observed in formulation (4) compared with formulation (2) suggested that increasing TA content could enhance the crosslinking density of the hydrogel network. Among all formulations, Formulation (5) and (6) showed the highest and comparable G′ values, indicating the strongest mechanical strength (Figure 2A). Considering that the aldehyde content of Formulation (6) was higher than that of Formulation (5), and free aldehyde groups may cause tissue toxicity, formulation (5) was selected for subsequent studies, in which OHA was 3 wt%, CMCS-PBA was 4 wt%, and TA was 0.2%. Unless otherwise stated, formulation (5) was used as the standard OHA-CP@TA hydrogel formulation for all subsequent characterization, in vitro biological assays, and in vivo experiments.

The hydrogel precursor solutions exhibited relatively low viscosity, which facilitated injection and rapid mixing (Figure S1). The gelation process of OHA-CP@TA hydrogel was visually validated using a vial inversion test, which appeared as a light brown hydrogel with uniform color (Figure 2D). And the gelation time was determined to be about 10 s (Figure 2B). FT-IR analysis showed the characteristic peaks of OHA, CMCS-PBA and TA in OHA-CP@TA hydrogel (Figure 2C). SEM micrographs revealed that the freeze-dried hydrogels possessed a highly interconnected porous architecture. Notably, treatment with H_2_O_2_ resulted in an increase in the pore size of the OHA-CP@TA hydrogel (Figure 2E), indicating that the hydrogel network exhibits ROS responsiveness, which was due to the formation of borate ester bonds.

### 3.3. Rheological and Self-Healing Properties of OHA-CP@TA Hydrogels

The rheological properties of the OHA-CP@TA hydrogel were analyzed using a rotational rheometer. OHA-CP and OHA-CP@TA hydrogels maintained G′ > G″ across a strain range of 0.1–30%, indicating solid-like viscoelastic behavior and a structurally stable network. However, because OHA-CP contains only a single crosslinked network, it exhibited a relatively narrower linear viscoelastic region (Figure 2G). Since the maximum strain encountered by human tissues is about 10% [31], the OHA-CP@TA hydrogel is anticipated to maintain structural integrity in physiological dynamic conditions. OHA-CP@TA hydrogel showed a significantly higher G′ value than that of OHA-CP hydrogel (Figure 2G,J), which was attributed to the formation of borate ester bonds between TA with PBA, thereby forming dual network cross-linking. Next, a frequency sweep test was conducted to further investigate the viscoelastic behavior. Across the frequency range of 0.1–10 Hz, the G′ values of both hydrogels remained consistently higher than their G″ values, confirming the maintenance of stable gel-like behavior. Notably, OHA-CP@TA exhibited a broader stable viscoelastic region (Figure 2H). Notably, this frequency window covers the physiological frequency range of human organs (4–6 Hz) [32], indicating that the OHA-CP@TA hydrogel is capable of retaining stable solid-like characteristics under in vivo physiological conditions. Further, shear-rate analysis was conducted to investigate the flow properties of the hydrogel that are critical for in vivo injection applications. As the shear rate increased from 0.1 to 1000 s^−1^, the viscosity of both hydrogels decreased by over four orders of magnitude (Figure 2I). This marked shear-thinning behavior endowed the hydrogels with favorable injectability, which was conductive for in vivo intrauterine injection. Further, the macroscopic injectability was confirmed by and extruded through a 22 G needle or a dropper with an inner diameter of 0.3 mm as continuous filaments (Figure 2F).

Finally, step-strain tests and step-shear-rate tests were performed to investigate the self-healing properties of the OHA-CP@TA hydrogel. At a low strain of 1%, the hydrogels exhibited strong elastic behavior (G′ > G″). In contrast, at a high strain of 500%, complete network destruction was observed (G′ < G″). Notably, the hydrogels exhibited excellent resilience, achieving over 95% modulus recovery over five consecutive loading cycles. However, after two cycles, the OHA-CP hydrogel failed to fully recover its initial modulus, indicating partial irreversible disruption of the network structure. This may be attributed to the absence of an effective secondary dynamic crosslinking network, resulting in a relatively fragile hydrogel network. In contrast, the incorporation of TA enhanced network stability and modulus recovery, further highlighting its advantage in improving the dynamic mechanical performance of the hydrogel (Figure 2K). Similarly, the shear-rate step tests, where shear rates were alternated between 1 s^−1^ and 100 s^−1^, revealed a comparable recovery trend (Figure 2L). Macroscopic self-healing behavior is displayed in Figure 2F, two semicircular hydrogel parts were brought into contact and allowed to rest, resulting in seamless interfacial fusion and diffusion of the dye. The self-healing capacity of the OHA-CP@TA hydrogel was ascribed to the dynamic association–dissociation of imine linkage and boronate bonds. These intrinsic self-healing properties endowed hydrogel with adaptability to the dynamically changing physiological microenvironment, thereby reinforcing its potential for intrauterine application.

### 3.4. In Vitro Swelling and Degradation of OHA-CP@TA Hydrogels

**Figure 3:**
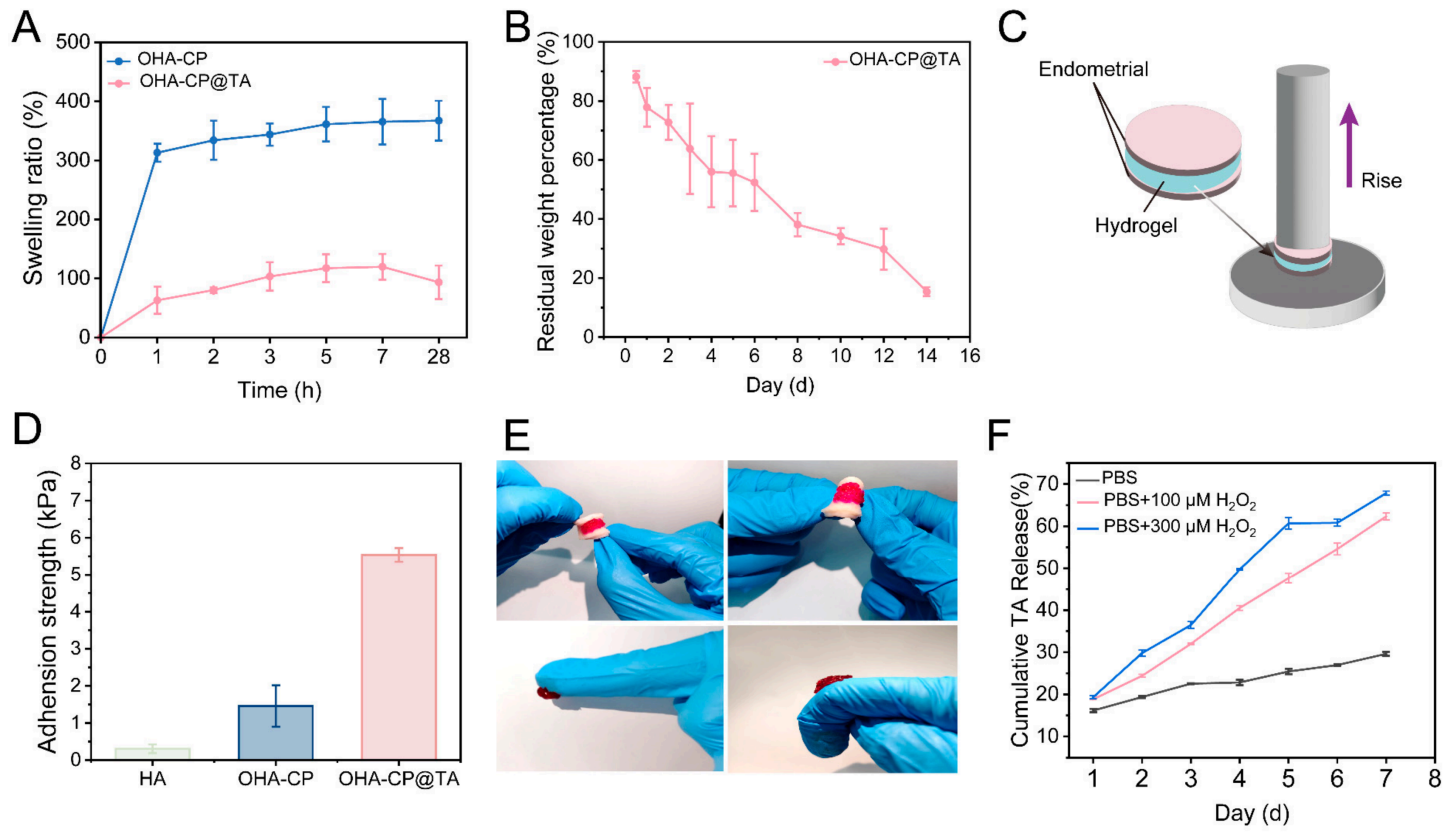
(**A**) The swelling ratio of OHA-CP@TA hydrogel at different time points. (**B**) In vitro degradation profiles of OHA-CP@TA hydrogel overtime. (**C**) Schematic illustration of adhesive force test and (**D**) quantitative analysis of adhesive strengths towards endometrial tissues. (**E**) Images of OHA-CP@TA adhering to porcine skin under movement. Adhesion ability of OHA-CP@TA hydrogels on gloves of fingers. (**F**) Cumulative release curves of TA from OHA-CP@TA hydrogel in PBS buffer containing different H_2_O_2_ concentration.

After immersion in uterine-simulating fluid (Table 2), the OHA-CP hydrogel exhibited rapid swelling behavior. The swelling ratio rapidly increased to approximately 320% within 1 h and gradually reached an equilibrium value of approximately 350% at 5 h. In contrast, OHA-CP@TA showed significantly reduced swelling, with swelling ratio slowly increasing to a maximum of ~100% at 5 h (Figure 3A). The lower water uptake of lyophilized OHA-CP@TA suggested a more compact network structure after TA incorporation, which may contribute to improved structural stability. Notably, the samples tested in this assay were not intended to mimic the actual clinical administration state. Instead, this assay was designed to characterize the hydration behavior of lyophilized hydrogels. Therefore, the results should be interpreted as the water-uptake properties of lyophilized hydrogel networks rather than the swelling behavior of freshly prepared injectable hydrogels.

The in vitro degradation profiles of hydrogels were evaluated by residual weight percentage over 14 days (Figure 3B). OHA-CP@TA hydrogel exhibited a time-dependent degradation trend, with residual weights declining to ~20% at day 14. The degradation of hydrogel avoids its secondary surgical removal after in vivo applications.

### 3.5. Tissue Adhesion of OHA-CP@TA Hydrogels

The tissue adhesion of OHA-CP@TA hydrogels towards endometrial tissue was detected (Figure 3C). OHA-CP@TA hydrogel exhibited an adhesion strength of approximately 5.5 kPa, which was significantly higher than that of OHA-CP hydrogel and commercial HA hydrogel (Figure 3D). The enhanced adhesion strength might be due to the formation of imine bonds, hydrogen bonds and phenylboronate bonds between tissues. Meanwhile, the OHA-CP@TA hydrogels could retain its original integrity and remain undamaged during bending manipulation on porcine skin (Figure 3E). The hydrogel could adhere to the finger regions of gloves and adapt well to finger movements, demonstrating good adhesive properties (Figure 3E). The above results indicated that OHA-CP@TA hydrogel has good tissue adhesion and is expected to remain at the application site and will not fall off with the movement of the organ.

### 3.6. ROS-Responsive Drug Release

As shown in Figure 3F, under physiological conditions, TA was released slowly and continuously, this sustained release pattern helps avoid rapid in vivo clearance. Therefore, 0, 100, and 300 μM H_2_O_2_ were used to establish an in vitro gradient from no oxidative stimulus to mild/moderate and stronger oxidative stress, allowing us to assess whether TA release increased with ROS levels. This ROS-responsive release is mainly attributed to the oxidation-sensitive dynamic boronate ester bonds between TA and PBA, enabling the hydrogel to regulate TA release according to local ROS levels at the injury site [33,34].

### 3.7. Cytocompatibility Evaluation

**Figure 4:**
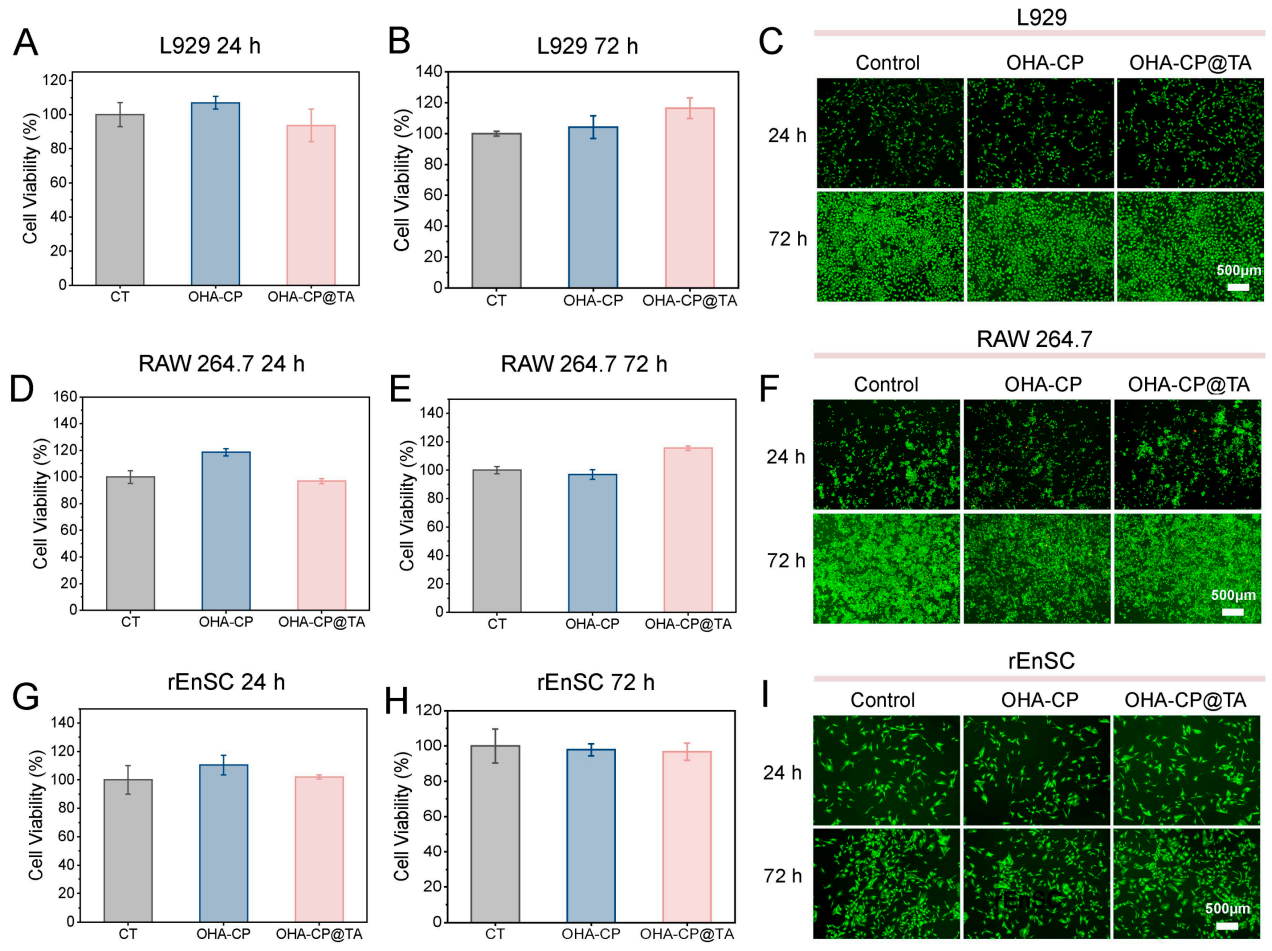
(**A**,**B**) Cell viability and (**C**) live/dead cell staining of L929 cells after treated with OHA-CP@TA hydrogel for 24 and 72 h. (**D**,**E**) Cell viability and (**F**) live/dead cell staining of RAW 264.7 cells after treated with OHA-CP@TA hydrogel for 24 and 72 h. (**G**,**H**) Cell viability and (**I**) live/dead cell staining of L929 cells after treated with OHA-CP@TA hydrogel for 24 and 72 h.

Initially, biocompatibility of precursor materials was assessed in RAW 264.7 and L929 cells. CMCS-PBA and OHA showed negligible cytotoxicity toward both cell lines when concentration below 0.625 mg/mL (Figure S2A–D). Next, cytocompatibility of hydrogels formed by precursor materials crosslinking was investigated in RAW 264.7 cells, L929 cells and rEnSCs. MTT results showed that the cell viability of RAW 264.7 cells, L929 cells and rEnSCs remained at >90% after 1 and 3 days of treatment with OHA-CP and OHA-CP@TA hydrogels, indicating no significant cytotoxicity (Figure 4A,B,D,E,G,H). Consistently, live/dead staining of RAW 264.7 and L929 cells for 1 day and 3 days revealed comparable cell counts and morphology in both hydrogels, further confirming the good biocompatibility of hydrogels (Figure 4C,F,I). The good cytocompatibility of the OHA-CP@TA hydrogel may be attributed to the fact that most of the aldehyde groups are cross-linked with the NH_2_ groups of CMCS, leaving a small amount of free aldehyde groups exposed.

### 3.8. Intracellular ROS Scavenging, Anti-Inflammatory and Macrophage Phenotype Regulation

Endometrial damage leads to excessive ROS production, which can trigger a pathological cascade including persistent inflammation and abnormal fibrosis [5]. These adverse processes disrupt normal tissue repair/regeneration, ultimately causing IUA. Thus, scavenging excess ROS and regulating inflammation are critical to preventing IUA.

To address this, we first evaluated the in vitro ROS scavenging activity of TA and OHA-CP@TA hydrogel against diverse ROS, including DPPH, •OH and ABTS radicals. VC, a classic antioxidant, was used as a positive control, and the scavenging performance was compared with that of free TA. The DPPH radical scavenging assay demonstrated that both TA and VC exhibited concentration-dependent scavenging capacities. When their concentrations reached ≥0.08 mg/mL, they showed comparable scavenging rates, with values exceeding 75% (Figure S3A).

**Figure 5:**
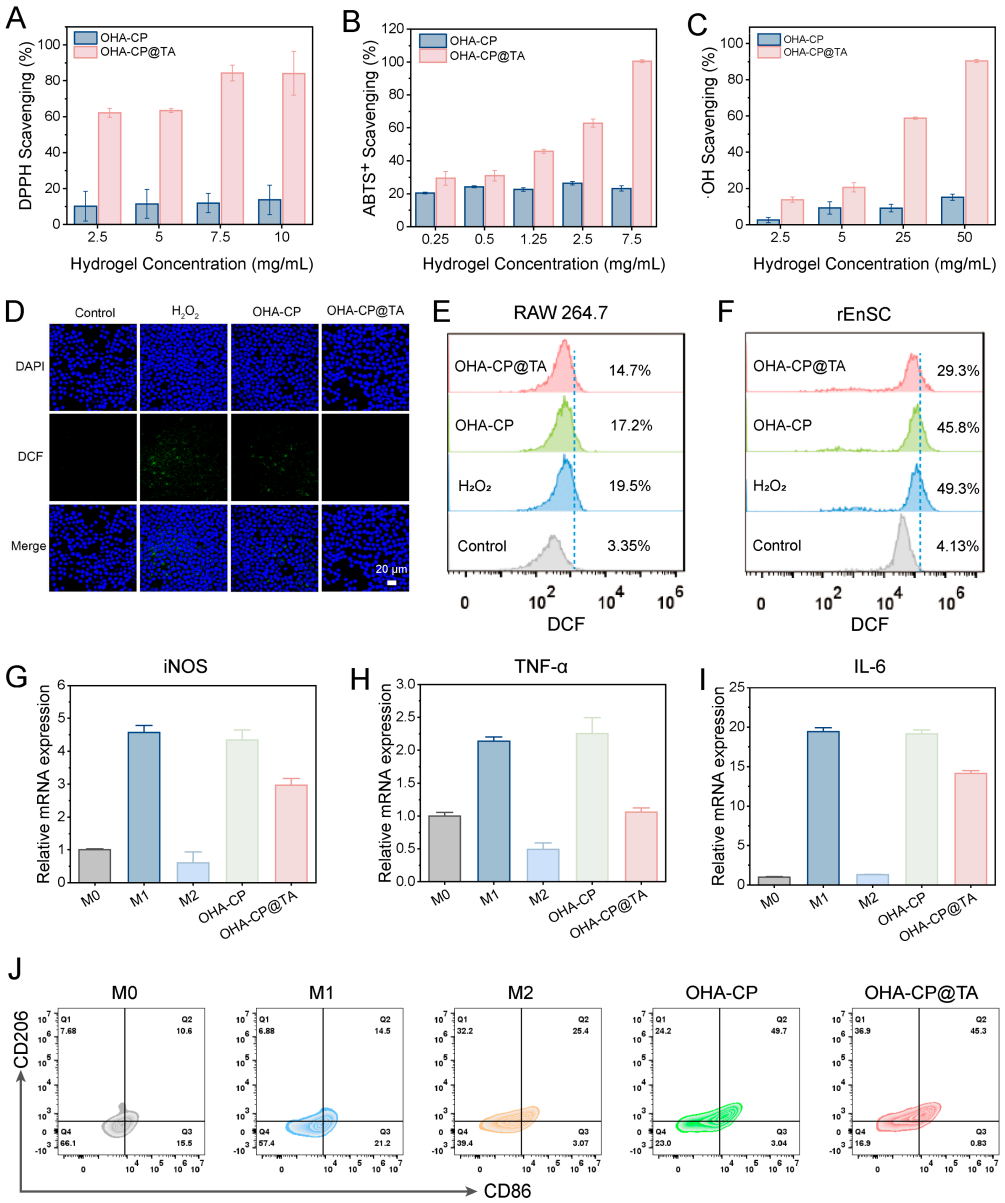
(**A**) DPPH scavenging rate, (**B**) ABTS+ scavenging rate, and (**C**) •OH scavenging rate of OHA-CP@TA hydrogels. (**D**) CLSM observation and (**E**) flow cytometry detection of intracellular ROS in H_2_O_2_-induced RAW 264.7 inflammatory cell models, respectively, following OHA-CP and OHA-CP@TA hydrogel extracts treatment. (**F**) Flow cytometry detection of intracellular ROS in H_2_O_2_-induced rEnSCs after treated with OHA-CP@TA hydrogel extracts. PT-PCR analysis of the mRNA expression of (**G**) iNOS, (**H**) TNF-α and (**I**) IL-6 in LPS-induced RAW 267.4 cells following OHA-CP@TA hydrogel extract treatment. (**J**) Representative photographs displaying CD86 and CD206 expression in LPS-induced RAW 267.4 cells treated with OHA-CP@TA hydrogel extracts.

Subsequently, OHA-CP and OHA-CP@TA hydrogels were selected for comparison to clarify the contribution of TA incorporation to the antioxidant and immunomodulatory functions of the hydrogel. The DPPH radical scavenging of hydrogels revealed that OHA-CP@TA displayed significantly enhanced scavenging efficiency compared with OHA-CP hydrogel, while OHA-CP hydrogel showed weaker scavenging activity, with a scavenging rate no more than 20% (Figure 5A). Next, the ABTS^+^ radicals scavenging assay showed that both TA and Vc exhibited concentration-dependent scavenging capacities, and TA’s scavenging activity was slightly stronger than that of Vc (Figure S3B). OHA-CP hydrogel displayed weaker scavenging activity against ABTS^+^ radicals, while OHA-CP@TA hydrogel showed concentration-dependent and significant enhanced scavenging activity (Figure 5B). By contrast, the •OH scavenging activity of TA was significantly stronger than that of Vc, and TA exhibited concentration-dependent scavenging capacities (Figure S2C). OHA-CP hydrogel displayed relatively weak scavenging activity against •OH radicals, while OHA-CP@TA hydrogel exhibited significant enhanced scavenging activity (Figure 5C). The above results suggested that TA has robust ROS-scavenging capacity, which is the main reason for the significantly enhanced ROS scavenging activity of OHA-CP@TA hydrogel. The certain ROS scavenging activity of OHA-CP hydrogel is presumably attributed to the phenylboronic acid moieties in the hydrogel matrices. The concentrations on the x-axis refer to the final mass concentrations of hydrogels in the reaction system for OHA-CP and OHA-CP@TA groups. For free TA and VC groups, the concentrations refer to the final concentrations of the tested compounds.

Subsequently, the intracellular ROS scavenging effect of the hydrogels was investigated in a H_2_O_2_-induced RAW 267.4 inflammatory cell model and an rEnSCs model, and analyzed by CLSM and flow cytometry using a DCFH-DA probe. H_2_O_2_ treatment (Figure 5D–F) induced increased DCF fluorescence, while treatment by OHA-CP@TA hydrogel extract at 125 nM TA equivalent reduced fluorescence intensity, indicating that the TA released from hydrogel could eliminate intracellular ROS. The ROS scavenging of OHA-CP@TA hydrogel in rEnSCs cells exhibited a similar result (Figure 5D–F).

Further, the anti-inflammatory capacity of hydrogel was investigated. RAW 264.7 cells treated with LPS+IFN-γ and IL-4 served as M1 macrophages and M2 macrophages, respectively. Cells without cytokine treatment were regarded as M0 macrophages. RT-PCR results revealed that free TA could markedly reduce the mRNA levels of pro-inflammatory markers (iNOS, TNF-α, and IL-6) over a wide range of concentrations (Figure S4). Meanwhile, compared with pro-inflammatory M1 macrophages, the mRNA expression of the pro-inflammatory markers (iNOS, TNF-α, and IL-6) was significantly downregulated in cells treated with OHA-CP@TA hydrogel extracts (Figure 5G). Furthermore, OHA-CP@TA hydrogel extract at 125 nM TA equivalent reduced the expression of CD86, while increasing the expression of CD206, suggesting it could promote M1 to M2 macrophage phenotype regulation (Figure 5F).

After endometrial injury, excessive ROS not only directly induces cellular oxidative damage, but also acts as an upstream signal to activate inflammation-related pathways, thereby promoting the expression of pro-inflammatory mediators such as TNF-α, IL-6, and iNOS and maintaining macrophage polarization toward the M1 phenotype. These results suggest that TA released from OHA-CP@TA hydrogel exerts multiple beneficial effects: it not only scavenges ROS and alleviates oxidative stress, but also suppresses inflammatory responses and promotes macrophage polarization toward the M2 phenotype, thereby providing a more favorable immune microenvironment for endometrial repair [5,35].

### 3.9. Hemocompatibility, In Vivo Retention, Degradation and Safety Evaluation

**Figure 6:**
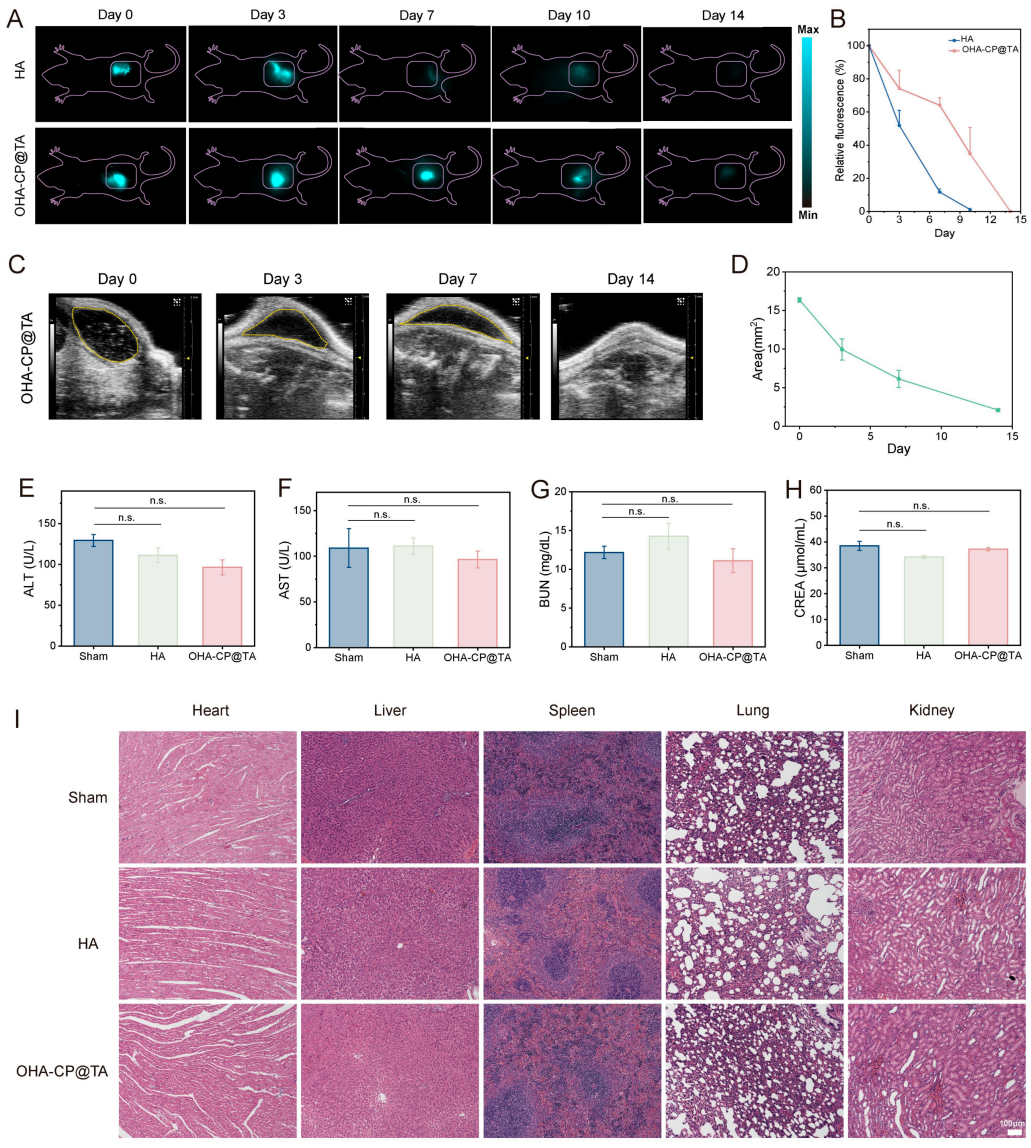
(**A**) NIRF-II imaging of different Cy7.5-labeled hydrogels in left uterine of rats and (**B**) the relative quantitative fluorescence area of different groups (*n* = 3). (**C**) Representative B-mode images and (**D**) the relative quantitative residual area of hydrogel recorded by ultrasound at different times. (The yellow region indicates the subcutaneously retained hydrogel.) (**E**–**H**) Blood biochemical index (ALT, AST, BUN and CREA) and (**I**) H&E staining of major organs after treated by OHA-CP@TA hydrogel. Scale bar: 100 μm. n.s. indicates no significant difference.

The hemolysis rates of OHA-CP@TA hydrogels were far below 5% (critical threshold of hemolysis), suggesting favorable hydrogel hemocompatibility (Figure S5). In this section, commercial HA hydrogel and the final candidate OHA-CP@TA hydrogel were compared to evaluate the improved intrauterine retention of OHA-CP@TA relative to the clinically used HA hydrogel. The retention of OHA-CP@TA hydrogel in rat uterine was evaluated by in vivo fluorescence imaging. NIRF-II imaging revealed that commercial HA hydrogel displayed nearly no measurable fluorescent at day 7, while OHA-CP@TA showed obvious fluorescent at day 7 and negligible fluorescent at day 14 (Figure 6A,B). The results demonstrated that OHA-CP@TA had a favorable in vivo retention capability compared with HA hydrogel, which might be attributed to its superior tissue adhesion and mechanical strength. Subsequently, the in vivo degradation of OHA-CP@TA hydrogel was investigated by monitoring the residual hydrogel area subcutaneously injected using ultrasound imaging. The hydrogel area exhibited a decrease over the observation period, becoming merely undetectable at day 14, suggesting a good degradation profile (Figure 6C,D). It is accepted that the first 0–7 days after surgery are the critical period for endometrial repair, and OHA-CP@TA can persist at the surgical site for at least a minimum of 7 days to cover the critical healing process and then it is degraded to obviate the requirement for secondary surgical removal, demonstrating an ideal hydrogel barrier for IUA prevention.

Additionally, the potential organ toxicity of OHA-CP@TA hydrogels was evaluated by blood biochemical analysis and histopathological examination of major organs. Liver function indexes (ALT, AST) and kidney function indexes (CREA, BUN) showed negligible differences among the sham group, HA hydrogel and OHA-CP@TA hydrogel group (Figure 6E–H). Concurrently, histopathological examination displayed no obvious abnormalities in major organs after OHA-CP@TA hydrogel treatment (Figure 6I). Collectively, these results suggested that the OHA-CP@TA hydrogel has favorable in vivo biosafety, with no detectable systemic toxicity or adverse impacts on the function of vital organs.

### 3.10. Therapeutic Efficacy and Mechanism of OHA-CP@TA Hydrogels

**Figure 7:**
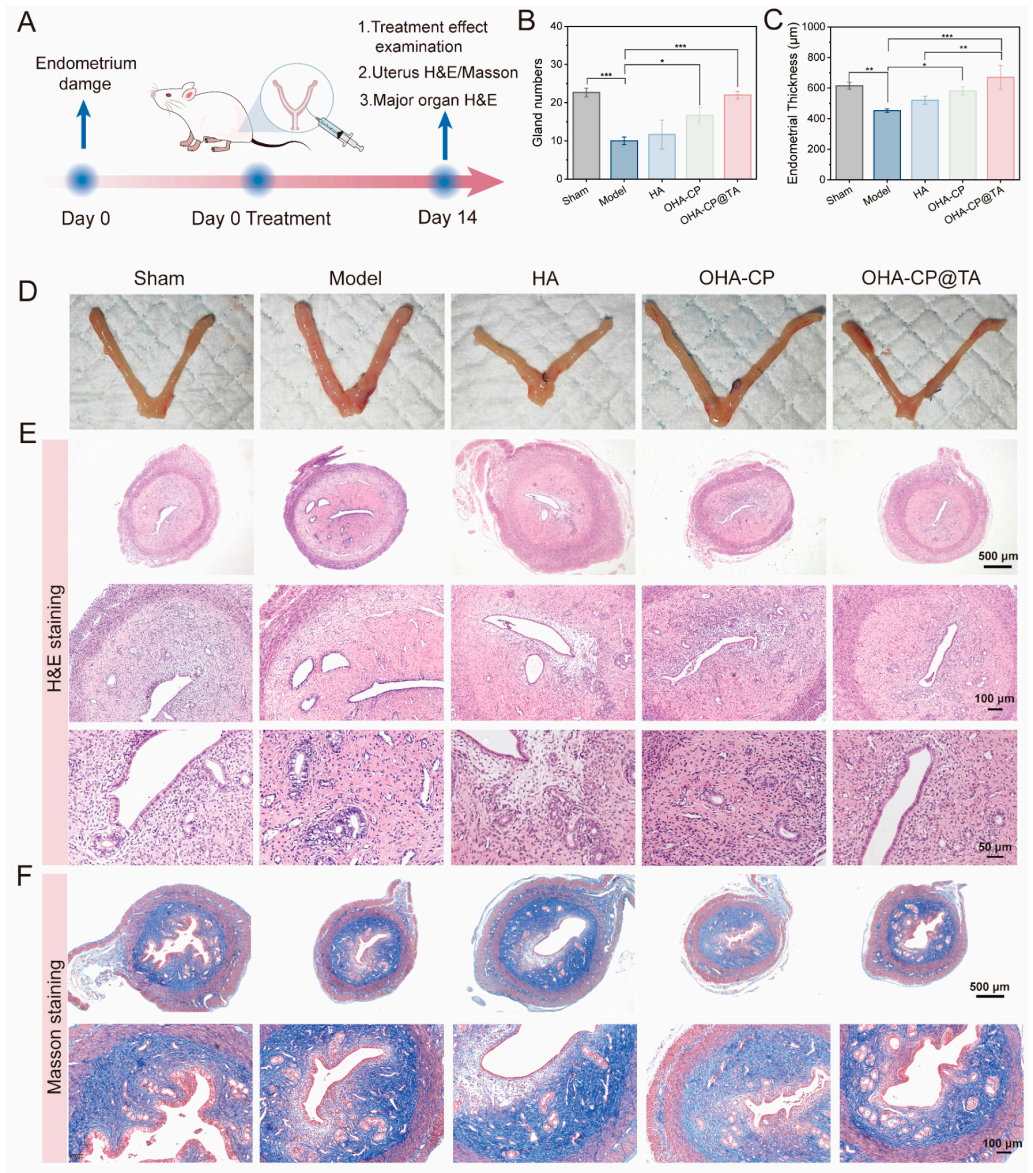
(**A**) Schematic illustration of experimental design of in vivo IUA prevention. (**B**) Gland numbers and (**C**) endometrial thickness in endometrium after different treatment at day 14. (*n* = 3) (**D**) Representative images of uterine tissues in different groups harvested at postoperative day 14. (**E**) H&E staining and (**F**) Masson’s trichrome staining after different treatment at day 14. * *p* < 0.05, ** *p* < 0.01, *** *p* < 0.001 (*n* = 3).

Given that most IUA are caused by endometrial damage during intrauterine manipulation, a rat endometrial curettage model was established to evaluate the anti-adhesion effect and endometrial repair ability of OHA-CP@TA hydrogel after surgery (Figure 7A). After 14 days of treatment, the uteri were collected for gross observation and sectioned for H&E and Masson staining, respectively. In this section, commercial HA, OHA-CP, and OHA-CP@TA groups were included to systematically compare the therapeutic effects of a clinically used barrier material, the TA-free hydrogel backbone, which mainly acts as a physical barrier, and the TA-functionalized hydrogel, which combines physical barrier function with microenvironment regulation. Gross observation of the uterine structures exhibited hyperemia and edema in the model group, after treatment by OHA-CP@TA hydrogel, the uterine morphology recovered towards a normal appearance (Figure 7D), and no hydrogel residue was seen in the uterine cavity. H&E staining results showed incomplete endometrial epithelial regeneration in the IUA model group, with disorganized cell arrangement and partial functional layer exposure, demonstrating typical features of adhesions (Figure 7E). Epithelial repair improved in the commercially available HA and OHA-CP groups compared with the model group, but mild defects or disorganized cell layers were still observed, suggesting that a purely physical barrier is limited. The commercially cross-linked HA hydrogel and OHA-CP groups exhibited improved epithelial repair versus the model group but retained mild cell layer disorganization, indicating limited efficacy of only passive physical barriers. In contrast, the OHA-CP@TA group displayed continuous, intact epithelium with regular morphology, confirming its superior ability to promote repair (Figure 7E). Subsequently, glandular morphology and quantity, and endometrial thickness which are important indicators reflecting endometrial repair were compared. The model group had ~10 glands, markedly fewer than the sham-operated group (~23.3 glands). OHA-CP@TA treatment restored glandular number to ~22 glands (comparable to sham) with normal morphology and intact structure, while the model and commercial HA groups showed irregular glands with partial atresia or cystic degeneration (indicative of impaired function) (Figure 6B). Additionally, the endometrial thickness in the model group (452.4 ± 11.3 μm) was significantly thinner than the sham group (615.9 ± 23.3 μm), whereas OHA-CP@TA treatment restored thickness to 670.3 ± 77.8 μm (Figure 6C). Masson’s trichrome staining showed that the collagen fibers were distributed as a fine reticular network in the sham group, while they were markedly dense and coarse in the model and HA groups. Collagen fibers in the OHA-CP group were significantly reduced compared to the HA group, mainly distributed as a fine reticular network, and the area of dense accumulation was markedly diminished, suggesting that adhesive barriers could reduce tissue fibrosis to some extent. By contrast, the collagen fibers in the OHA-CP@TA group were distributed sparsely and slenderly, resembling the physiological collagen network, indicating that OHA-CP@TA could effectively suppress pathological fibrosis and promote tissue repair (Figure 7F).

**Figure 8:**
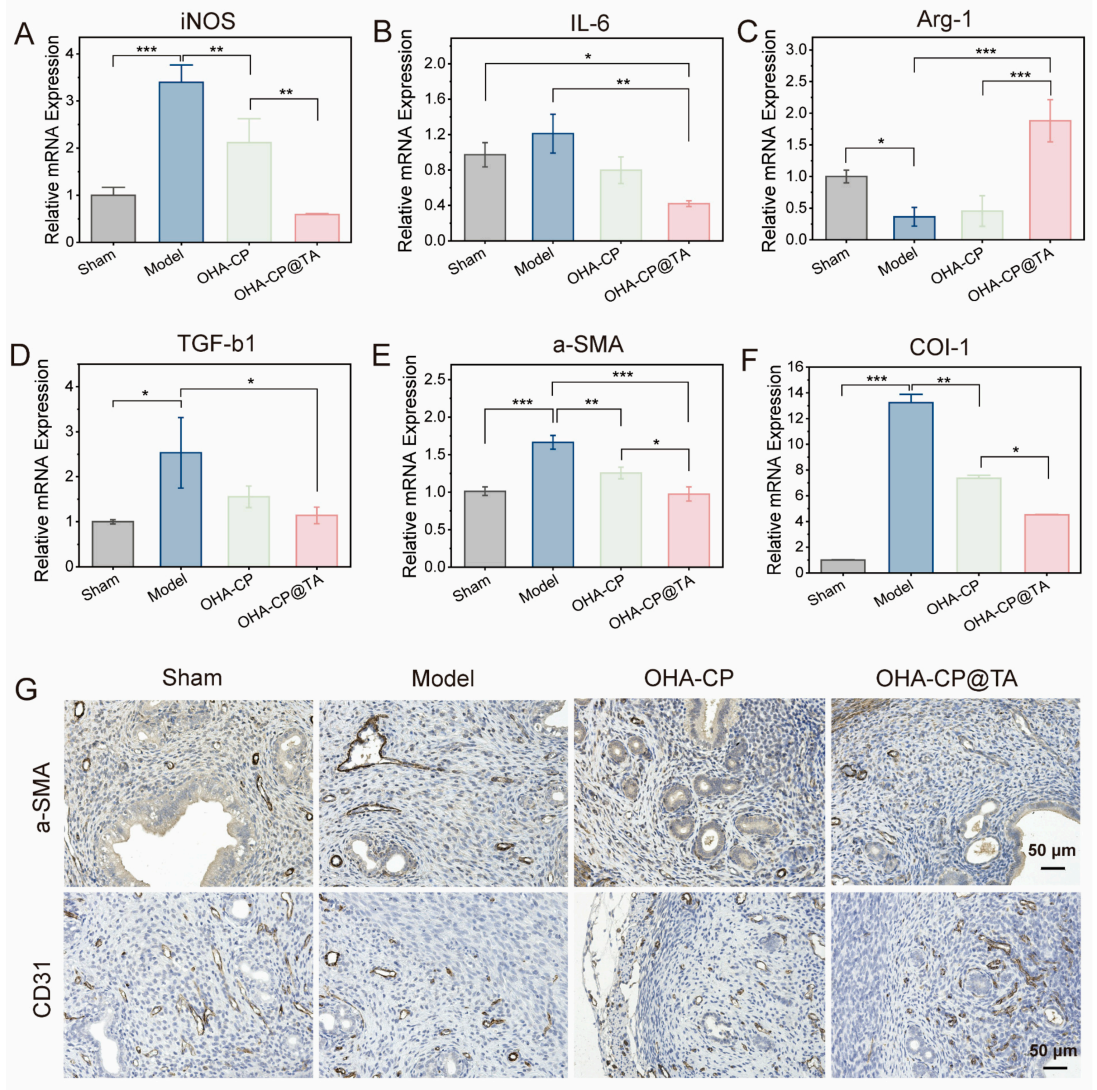
(**A**–**F**) Relative mRNA expressions levels of iNOS, IL-6, Arg-1, TGF-β1, α-SMA and Col-1, and (**G**) immunohistochemical staining for α-SMA and CD31, in uterine tissues after different treatment. Scale bar: 50 μm. * *p* < 0.05, ** *p* < 0.01, *** *p* < 0.001 (*n* = 3).

Post-endometrial injury, persistent or dysregulated inflammation is a key driver of IUA formation, as increased inflammation and oxidative stress can upregulate TGF-β1 and thereby promote subsequent fibrosis. Further, the therapeutic mechanism of OHA-CP@TA hydrogel in preventing IUA was investigated by RT-PCR using collected uterine tissues after treatment. As shown in Figure 8A–C, the mRNA expression of iNOS and IL-6 in OHA-CP@TA hydrogel-treated groups were significantly reduced, while the mRNA expression of Arg-1 was dramatically increased, confirming its robust capacity to suppress inflammatory responses and promote M2 macrophage polarization to promote repair. Additionally, fibrosis-related factors, including TGF-β1, α-SMA and Col-1 were analyzed. As displayed in Figure 8D–F, TGF-β1, α-SMA, and Col-1 showed reduced expression in the OHA-CP@TA hydrogel-treated group compared to the Model and OHA-CP groups, indicating OHA-CP@TA could prevent endometrium fibrosis. Further, immunohistochemical staining for α-SMA (a key driver of postoperative fibrosis) and CD31 (an endothelial marker, indicative of neovascularization) was performed. The OHA-CP@TA-treated group exhibited significantly reduced α-SMA-positive myofibroblast accumulation compared with the model group, suggesting the inhibition of myofibroblast activation (Figure 8G). Correspondingly, CD31 staining revealed enhanced and more orderly CD31-positive vascular structures in the hydrogel-treated groups, indicating that the hydrogel system promoted favorable neovascularization, which supports nutrient exchange and tissue regeneration at the repair site (Figure 8G).

Overall, OHA-CP@TA hydrogel can scavenge excessive local ROS through ROS-responsive TA release. Reduced ROS levels may suppress pro-inflammatory signaling, decrease iNOS and IL-6 expression, and promote macrophage transition from the M1 phenotype toward the repair-associated M2 phenotype. Meanwhile, inflammation attenuation may reduce TGF-β-related fibrotic stimulation, thereby downregulating α-SMA and Col-1 expression and inhibiting abnormal ECM deposition. The resulting low-oxidative and low-inflammatory microenvironment favors endometrial cell survival, angiogenesis, and glandular regeneration, as reflected by increased endometrial thickness, enhanced CD31-positive vessels, and restored gland numbers.

The above results suggested that OHA-CP@TA hydrogel has satisfactory therapeutic effects in promoting endometrial regeneration and preventing the formation of IUA through physical barriers and intelligent inflammation regulation.

## 4. Conclusions

In summary, we successfully engineered a ROS-responsive and bioadhesive injectable hydrogel, OHA-CP@TA, which effectively prevented IUA and promoted endometrial regeneration. This dual-crosslinked system was demonstrated to exhibit ideal physical properties for intrauterine application, including good mechanical strength, injectability, self-healing, shear-thinning behaviors, and crucially, robust and sustained adhesion to uterine tissues, enabling effective in vivo retention over at least 7 days. Within the postoperative inflammatory environment, TA was released in a ROS-dependent manner, and the released TA acts as a potent scavenger of multiple ROS, effectively reducing local inflammation and promoting the polarization of M1 macrophages towards the reparative M2 phenotype. This targeted ROS scavenging and immunomodulation further inhibited endometrial fibrosis progression, as evidenced by downregulation of α-SMA and Col-1 expression, and actively promotes comprehensive endometrial regeneration, demonstrated by enhanced angiogenesis, increased endometrial thickness, and restoration of glandular numbers. Additionally, OHA-CP@TA demonstrated appropriate in vivo biodegradability and safety profiles. Collectively, these findings demonstrated that by providing a sustained physical barrier while simultaneously modulating the hostile inflammatory and oxidative microenvironment, OHA-CP@TA effectively prevented IUA and promoted functional endometrial repair. Therefore, the current design represents a highly promising and clinically translatable strategy for significantly improving the management of IUA.

## Figures and Tables

**Table 1:** Formulations of OHA-CP@TA hydrogels.

Formulations	CMCS-FPBA (wt%)	OHA (wt%)	TA (wt%)
(1)	3	2	0.2
(2)	3	3	0.2
(3)	4	2	0.1
(4)	4	2	0.2
(5)	4	3	0.2
(6)	4	4	0.2

## Data Availability

Original datasets are available from the corresponding author on request.

## Supplementary Materials

The following supporting information can be downloaded at: https://www.mdpi.com/article/10.3390/pharmaceutics18060685/s1, Figure S1. Viscosity profiles of hydrogel precursor solutions as a function of shear rate. Figure S2. (**A**–**D**) Cell viability of CMCS-PBA and OHA towards L929 cells and RAW264.7 cells after treated with various concentrations for 24 and 72 h. Figure S3. (**A**) DPPH scavenging rate, (**B**) ABTS+ scavenging rate, and (**C**) •OH scavenging rate of different concentration of free TA and Vc. Figure S4. PT-PCR analysis of the mRNA expression of iNOS, TNF-α, and IL-6 in LPS-induced RAW 264.7 cells following treatment with different concentration of free TA. Figure S5. The hemolysis behavior of OHA-CP@TA hydrogel with different volume. Table S1. The sequences of primers used in RT-PCR.

## References

[1] Wen L., Liu Q., Xu J., Liu X., Shi C., Yang Z., Zhang Y., Xu H., Liu J., Yang H. (2020). Recent advances in mammalian reproductive biology. Sci. China Life Sci..

[2] Di Guardo F., Della Corte L., Vilos G.A., Carugno J., Török P., Giampaolino P., Manchanda R., Vitale S.G. (2020). Evaluation and treatment of infertile women with Asherman syndrome: An updated review focusing on the role of hysteroscopy. Reprod. Biomed. Online.

[3] Healy M.W., Schexnayder B., Connell M.T., Terry N., DeCherney A.H., Csokmay J.M., Yauger B.J., Hill M.J. (2016). Intrauterine adhesion prevention after hysteroscopy: A systematic review and meta-analysis. Am. J. Obstet. Gynecol..

[4] Zhang W., Wu H., Xie L., Liang Y., Huang X., Yang Z., Xu T., Lin F. (2024). A two-component peptide-based hydrogel for endometrial repair and restoring fertility. Chin. Chem. Lett..

[5] Qin Z., Yu Q., Long Y. (2025). Unveiling the pathological landscape of intrauterine adhesion: Mechanistic insights and exosome-biomaterial therapeutic innovations. Int. J. Nanomed..

[6] Ang C.J., Skokan T.D., McKinley K.L. (2023). Mechanisms of regeneration and fibrosis in the endometrium. Annu. Rev. Cell Dev. Biol..

[7] Qin X., Hu K., Li Q., Sun Y., Peng T., Liu X., Li J., Nan W., Yu Y., Qi X. (2025). In situ sprayed hydrogel delivers extracellular vesicles derived from human endometrial organoids for uterine function preservation and fertility restoration. Adv. Healthc. Mater..

[8] Kou L., Jiang X., Xiao S., Zhao Y.-Z., Yao Q., Chen R. (2020). Therapeutic options and drug delivery strategies for the prevention of intrauterine adhesions. J. Control. Release.

[9] Wang J., Chen Q., Wang Y., Gan Z., Wu M., Shang L., Duan P. (2025). Multiresponsive Microcapsules for Prevention of Intrauterine Adhesion. ACS Nano.

[10] Leung R.K.-K., Lin Y., Liu Y. (2021). Recent advances in understandings towards pathogenesis and treatment for intrauterine adhesion and disruptive insights from single-cell analysis. Reprod. Sci..

[11] Cao Y., Wu J., Huang J., Fan X., Zhang Y., Li L., Dai Y. (2025). Human embryonic stem cell-derived immunity-and-matrix-regulatory cells promote endometrial repair and fertility restoration in IUA rats. Stem Cell Res. Ther..

[12] Wang L., Yu C., Chang T., Zhang M., Song S., Xiong C., Su P., Xiang W. (2020). In situ repair abilities of human umbilical cord–derived mesenchymal stem cells and autocrosslinked hyaluronic acid gel complex in rhesus monkeys with intrauterine adhesion. Sci. Adv..

[13] Wang B., Feng C., Dang J., Zhu Y., Yang X., Zhang T., Zhang R., Li J., Tang J., Shen C. (2020). Preparation of fibroblast suppressive poly (ethylene glycol)-b-poly (l-phenylalanine)/poly (ethylene glycol) hydrogel and its application in intrauterine fibrosis prevention. ACS Biomater. Sci. Eng..

[14] Xiao S., Wan Y., Xue M., Zeng X., Xiao F., Xu D., Yang X., Zhang P., Sheng W., Xu J. (2014). Etiology, treatment, and reproductive prognosis of women with moderate-to-severe intrauterine adhesions. Int. J. Gynecol. Obstet..

[15] Zhang M., Zhang H., Zhang P., Wei Z., Liao X. (2025). The Efficacy of Self-Cross-Linked Sodium Hyaluronate Gel in the Prevention of Intrauterine Adhesions: A Literature Review. Clin. Exp. Obstet. Gynecol..

[16] Cai G., Hou Z., Sun W., Li P., Zhang J., Yang L., Chen J. (2022). Recent Developments in Biomaterial-Based Hydrogel as the Delivery System for Repairing Endometrial Injury. Front. Bioeng. Biotechnol..

[17] Feng L., Wang L., Ma Y., Duan W., Martin-Saldaña S., Zhu Y., Zhang X., Zhu B., Li C., Hu S. (2023). Engineering self-healing adhesive hydrogels with antioxidant properties for intrauterine adhesion prevention. Bioact. Mater..

[18] Sun W., Xu W., Xiao M., Zhang X., Chen J., Zhang J., Yang L., Na Q. (2025). Enhancing exosome stability and delivery with natural polymers to prevent intrauterine adhesions and promote endometrial regeneration: A review. J. Nanobiotechnol..

[19] Xu H.-L., Xu J., Shen B.-X., Zhang S.-S., Jin B.-H., Zhu Q.-Y., ZhuGe D.-L., Wu X.-Q., Xiao J., Zhao Y.-Z. (2017). Dual regulations of thermosensitive heparin–poloxamer hydrogel using ε-polylysine: Bioadhesivity and controlled KGF release for enhancing wound healing of endometrial injury. ACS Appl. Mater. Interfaces.

[20] Wu J., Deng J., Theocharidis G., Sarrafian T.L., Griffiths L.G., Bronson R.T., Veves A., Chen J., Yuk H., Zhao X. (2024). Adhesive anti-fibrotic interfaces on diverse organs. Nature.

[21] Cai H., Wang J., Zheng Y., Wen J., Li H. (2025). Biocompatible injectable glycyrrhizic acid-based growth factor hydrogel for synergistic endometrial regeneration. Mater. Today Bio.

[22] Chen X., Zhu C., Wang L., Deng J., Wang Z., Wu X., Tan H., Qu Y., Wu B., Li Y. (2025). Autologous platelet-rich plasma dual-network hydrogel promotes human endometrial regeneration. Acta Biomater..

[23] Zheng X., Huang R., Yin L., Yao M., Chu J., Yang F., Dong Y., Zhao M., Ma T. (2025). Injectable antioxidant hyaluronan/chitosan hydrogel as a platelet-rich plasma and stem cell carrier to promote endometrial regeneration and fertility restoration. Acta Biomater..

[24] Ji W., Wen J., Tong N., Guo F., Zheng J., Wen X., Liu J., Zhang N., Hou B. (2026). A pH-responsive hyaluronic acid hydrogel facilitates lesion-localized integrin α5β1 agonism to attenuate osteopontin signaling and fibrosis in intrauterine adhesions. Acta Biomater..

[25] Fakhari A., Berkland C. (2013). Applications and emerging trends of hyaluronic acid in tissue engineering, as a dermal filler and in osteoarthritis treatment. Acta Biomater..

[26] Martău G.A., Mihai M., Vodnar D.C. (2019). The use of chitosan, alginate, and pectin in the biomedical and food sector—Biocompatibility, bioadhesiveness, and biodegradability. Polymers.

[27] Guan X., Zhang B., Wang Z., Han Q., An M., Ueda M., Ito Y. (2023). Natural polyphenol tannin-immobilized composites: Rational design and versatile applications. J. Mater. Chem. B.

[28] Zhao L.-L., Luo J.-J., Cui J., Li X., Hu R.-N., Xie X.-Y., Zhang Y.-J., Ding W., Ning L.-J., Luo J.-C. (2024). Tannic acid-modified decellularized tendon scaffold with antioxidant and anti-inflammatory activities for tendon regeneration. ACS Appl. Mater. Interfaces.

[29] Huang Y., Dai X., Gong Y., Ren L., Luo Y., Sun Y., Chen M., Jiang J., Guan Z., Zhao C. (2024). ROS-responsive sprayable hydrogel as ROS scavenger and GATA6+ macrophages trap for the prevention of postoperative abdominal adhesions. J. Control. Release.

[30] Zhang C., Xu N., Yang B. (1996). The corrosion behaviour of copper in simulated uterine fluid. Corros. Sci..

[31] Stapleton L.M., Steele A.N., Wang H., Hernandez H.L., Yu A.C., Paulsen M.J., Smith A.A.A., Roth G.A., Thakore A.D., Lucian H.J. (2019). Use of a supramolecular polymeric hydrogel as an effective post-operative pericardial adhesion barrier. Nat. Biomed. Eng..

[32] Randall J.M., Matthews R.T., Stiles M.A. (1997). Resonant frequencies of standing humans. Ergonomics.

[33] Zhu Y., Song Z., Liu B., Gao A., Tian F., Tian H. (2026). Platelets reduce oxidative stress damage in human endometrial stromal cells in vitro. Reprod. Biomed. Online.

[34] Zhao F., Wei W., Huang D., Guo Y. (2023). Knockdown of miR-27a reduces TGFβ-induced EMT and H_2_O_2_-induced oxidative stress through regulating mitochondrial autophagy. Am. J. Transl. Res..

[35] Stewart A.G., Thomas B., Koff J. (2018). TGF-β: Master regulator of inflammation and fibrosis. Respirology.

