# Supplementary material for: A Reactive Oxygen Species-Responsive Biomimetic Adhesive Hydrogel Mediates Immunoregulation to Effectively Prevent Intrauterine Adhesions
Source: Pharmaceutics. 2026 May 30;18(6):685. doi: 10.3390/pharmaceutics18060685 (PMC13306208; doi:10.3390/pharmaceutics18060685)
Supplement: Supplementary file 1 [file pharmaceutics-18-00685-s001.zip › pharmaceutics-4278465-supplementary.pdf]

Supplementary materials: A Reactive Oxygen Species-Responsive Biomimetic Adhesive Hydrogel Mediates Immunoregulation to Effectively Prevent Intrauterine Adhesions

Wanzhen Li, Chenyu Liao, Yuzhen Li, Zijun Lin, Danni Xiao, Gengsheng Ye, Yanjuan Huang, Chunshun Zhao and Shengmiao Cui

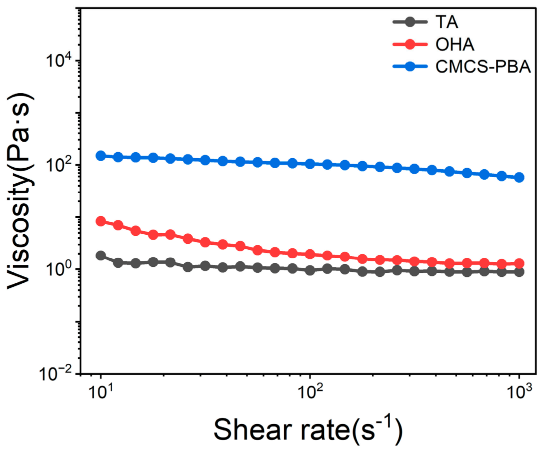

Figure S1. Viscosity profiles of hydrogel precursor solutions as a function of shear rate.

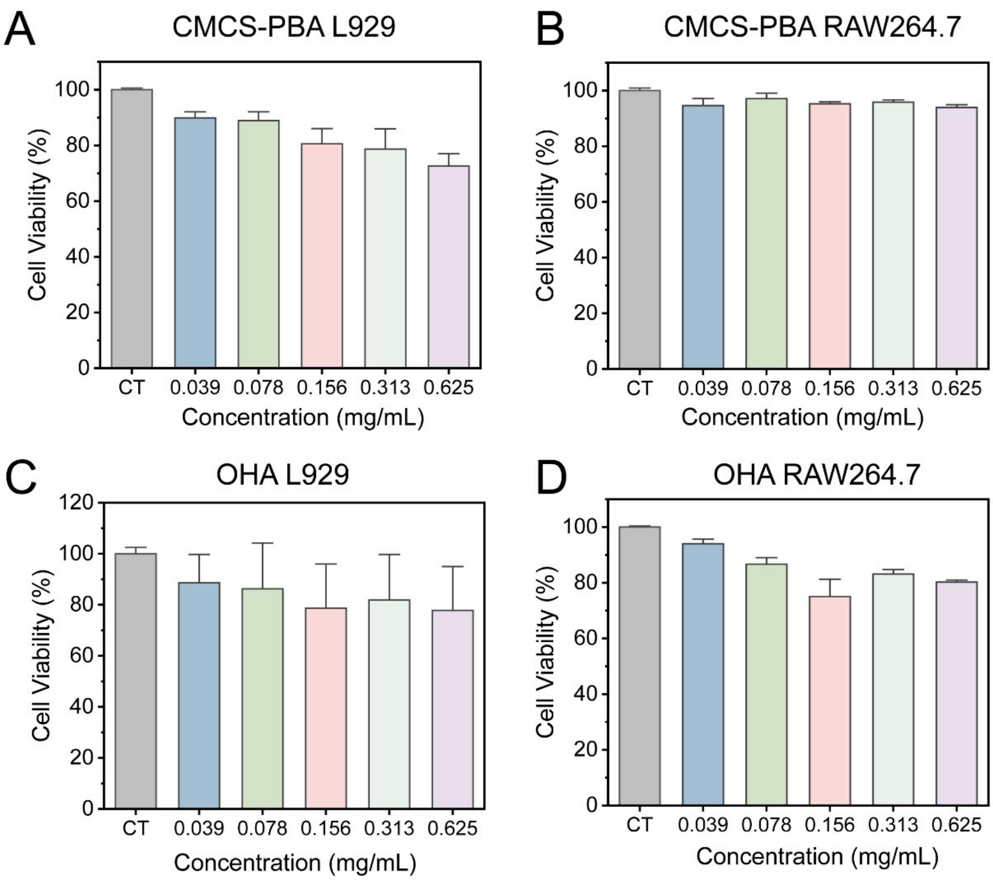

Figure S2. (A-D) Cell viability of CMCS-PBA and OHA towards L929 cells and RAW264.7 cells after treated with various concentrations for 24 and 72 h.

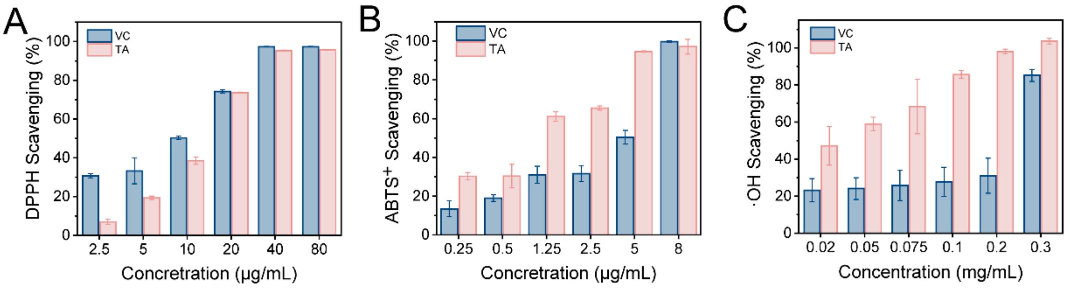

Figure S3. (A) DPPH scavenging rate, (B) ABTS+ scavenging rate, and (C) •OH scavenging rate of different concentration of free TA and Vc.

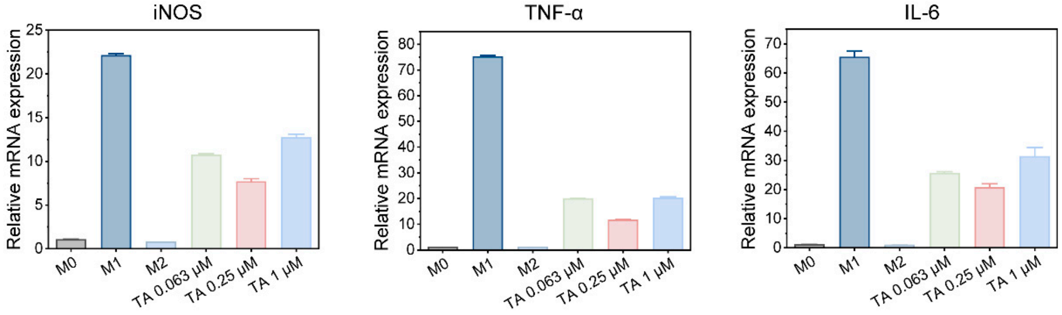

Figure S4. PT-PCR analysis of the mRNA expression of iNOS, TNF-α, and IL-6 in LPS-induced RAW 264.7 cells following treatment with different concentration of free TA.

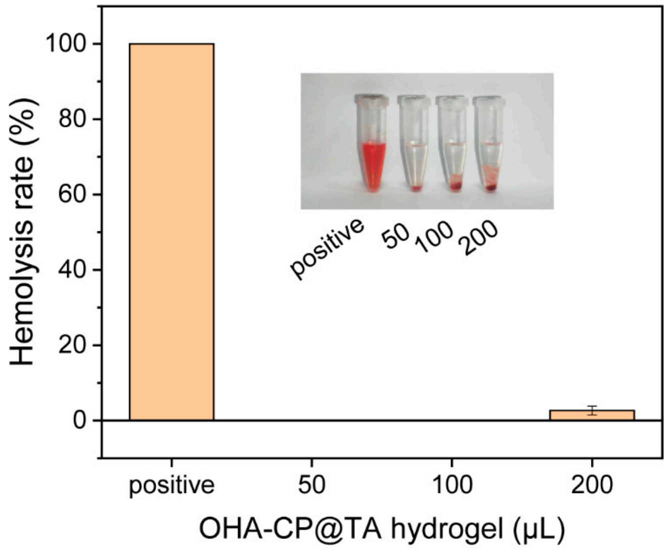

Figure S5. The hemolysis behavior of OHA-CP@TA hydrogel with different volume.

Table S1. The sequences of primers used in RT-PCR.

Primer	Sequence
GAPDH F (mouse)	TGACCTCAACTACATGGTCTACA
GAPDH R (mouse)	CTTCCCATTCTCGGCCTTG
iNOS F (mouse)	GAAGAAAACCCCTTGTGCTG
iNOS R (mouse)	TCCAGGGATTCTGGAACATT
TNF-α F (mouse)	TCTTCTCATTCCTGCTTGTGG
TNF-α R (mouse)	ATGAGAGGGAGGCCATTTG
IL-6 F (mouse)	TGTGCAAGTGTCTGAAGCAGC

IL-6 R (mouse)	TGGAAGCAGCCCTTCATCTT
GAPDH F (rat)	ACTTTGGTATCGTGGAAGGACT
GAPDH R (rat)	GTAGAGGCAGGGATGATGTTCT
iNOS F (rat)	TCAACTACAAGCCCCACGG
iNOS R (rat)	GAGAAACTTCCAGGGGCAAG
IL-6 F (rat)	TAGTCCTTCCTACCCCAATTTCC
IL-6 R (rat)	TTGGTCCTTAGCCACTCCTTC
Arg-1 F (rat)	CTCCAAGCCAAAGTCCTTAGAG
Arg-1 R (rat)	AGGAGCTGTCATTAGGGACATC
TGF-β1 F (rat)	CATTGCTGTCCCGTGCAGA
TGF-β1 R (rat)	AGGTAACGCCAGGAATTGTTGCTA
COI-1a F (rat)	CCCAGCGGTGGTTATGACTT
COI-1a R (rat)	TCGATCCAGTACTCTCCGCT
